# Plastid and mitochondrial genomes of *Coccophora langsdorfii* (Fucales, Phaeophyceae) and the utility of molecular markers

**DOI:** 10.1371/journal.pone.0187104

**Published:** 2017-11-02

**Authors:** Louis Graf, Yae Jin Kim, Ga Youn Cho, Kathy Ann Miller, Hwan Su Yoon

**Affiliations:** 1 Department of Biological Sciences, Sungkyunkwan University, Suwon, Korea; 2 National Institute of Biological Resources, Incheon, Korea; 3 University Herbarium, University of California, Berkeley, CA, United States of America; Agriculture and Agri-Food Canada, CANADA

## Abstract

*Coccophora langsdorfii* (Turner) Greville (Fucales) is an intertidal brown alga that is endemic to Northeast Asia and increasingly endangered by habitat loss and climate change. We sequenced the complete circular plastid and mitochondrial genomes of *C*. *langsdorfii*. The circular plastid genome is 124,450 bp and contains 139 protein-coding, 28 tRNA and 6 rRNA genes. The circular mitochondrial genome is 35,660 bp and contains 38 protein-coding, 25 tRNA and 3 rRNA genes. The structure and gene content of the *C*. *langsdorfii* plastid genome is similar to those of other species in the Fucales. The plastid genomes of brown algae in other orders share similar gene content but exhibit large structural recombination. The large in-frame insert in the *cox2* gene in the mitochondrial genome of *C*. *langsdorfii* is typical of other brown algae. We explored the effect of this insertion on the structure and function of the *cox2* protein. We estimated the usefulness of 135 plastid genes and 35 mitochondrial genes for developing molecular markers. This study shows that 29 organellar genes will prove efficient for resolving brown algal phylogeny. In addition, we propose a new molecular marker suitable for the study of intraspecific genetic diversity that should be tested in a large survey of populations of *C*. *langsdorfii*.

## Introduction

*Coccophora langsdorfii* (Turner) Greville is a brown algal species (Sargassaceae, Fucales) found in intertidal habitats along the cold waters of the Northeast Asia. Its range includes the East Sea in Korea [1], Hokkaido and the northern part of Japan [2], and the Far East coast of Russia and the Sakhalin Islands [3]. It forms dense bushy stands that stabilize the sandy sediment in which they grow and serve as support for epiphytic algae and associated herbivores. While the general morphology of the sporophyte is similar to species in the genus *Sargassum*, *C*. *langsdorfii* exhibits apical reproductive structures that clearly separate it from *Sargassum* species. Recent molecular phylogenetic studies demonstrated that *C*. *langsdorfii* is a close sister taxon to the genera *Bifurcaria* and *Cystoseira* within the family Sargassaceae [4–6].

In recent years, likely due to global climate change and/or habitat loss, *C*. *langsdorfii* populations in Korea have dramatically decreased and the species has been designated as an endangered species [7]. It has become urgent to establish coherent and efficient management for the populations of this alga. Conservation strategies and marine reserve design need to be based on knowledge of dispersal processes and gene flow among populations [8]. As sequencing technologies become less expensive and faster, it is possible to characterize such processes in wild populations [9]. In the genus *Sargassum*, *cox3*, a mitochondrial molecular marker, has been used to investigate population genetic structure [10]. Despite increasing interest in this kind of information, only a handful of population studies have been conducted on brown algae [10–12] and no such study is available for *C*. *langsdorfii*. In an effort to provide insight into the evolution of *C*. *langsdorfii* and to help develop robust conservation strategies, we sequenced the complete plastid genome (ptDNA) and mitochondrial genome (mtDNA) of this alga. Using this genomic information, we conducted a study of the utility for phylogenetic studies of the genes encoded in the ptDNA and mtDNA. We suggest that 29 organellar genes are informative in phylogenetic studies of brown algae. Furthermore, we demonstrate the utility of this genomic information as a source of molecular markers for elucidating population structure for conservation purposes.

## Materials and methods

### Field samples and DNA extraction

Fresh thalli of *Coccophora langsdorfii* were collected on 28 March 2015 from the shallow subtidal zone of Gonghyunjin, Gangwon-do, South Korea (38°21'22''N, 128°30'45''E). Thalli were washed in autoclaved seawater, dried with paper towels, and preserved in silica gel. Voucher specimens were deposited in the herbarium of Sungkyunkwan University (SKKU003999). Genomic DNA was extracted from 2 mg of dried material following the protocol of Sahu et al. [13] with a subsequent purification step using the PowerClean® DNA Clean-Up Kit (Qiagen, Carlsbad, CA).

### Whole-genome de novo sequencing and assembly

A 400 bp sequencing library was built using the IonXpress Plus gDNA Fragment Library Kit following manufacturer's instructions (Thermo Fisher Scientific, San Francisco, CA) and was sequenced using the Ion Torrent Personal Genome Machine (Thermo Fisher Scientific, San Francisco, CA). Organelle genome assemblies and annotations were summarized as in [14]. A total of 5,996,144 sequencing reads were assembled using MIRA assembler v4.0.2.1 [15] and the CLC de novo assembler included in CLC Genomics Workbench v5.5.1 (CLC Bio, Aarhus, Denmark). The contigs were sorted using a combination of BLAST [16] and Mummer plotting [17] with the plastid genome of *Fucus vesiculosus* (NC_016735) and the mitochondrial genome of *Sargassum thunbergii* (NC_026700) as references. Contigs identified as part of the plastid genome or the mitochondrial genome were independently assembled using Geneious Pro v7 [18], which generated one circular contig each. Ambiguous sequences were confirmed by Sanger sequencing (Macrogen Inc., Seoul, Korea). All sequencing reads were mapped on both sequences with an average coverage of 213X and 119X for the plastid and mitochondrial genomes, respectively. The complete plastid genome was annotated by using a combination of DOGMA [19] and BLASTx [16] for protein-coding sequences; tRNA scan-SE [20] for the tRNA sequences and RNAmmer [21] for ribosomal RNA sequences. All annotations were then manually checked. Finalized genome maps were created with GenomeVx [22].

### Gene content comparison for organelle genomes

All available plastid genomes of the Stramenopiles were downloaded from NCBI GenBank (https://www.ncbi.nlm.nih.gov/genbank/, consulted on 17 May 2016). A list of annotated genes was extracted and compared using newly written R scripts (R core team, 2012; https://www.R-project.org). Protein-coding genes not shared among all taxa were submitted to local BLASTp [16] search (*e-*value cut-off ≤10^−6^) against the other phaeophycean plastid genomes, with the result that some genes, especially those coding for hypothetical proteins, were annotated under different names. Based on those results, we propose a revised and unified annotation for the plastid genome of the class Phaeophyceae (S1 Table). Gene names of the extracted annotations were manually checked, and unmatched names were modified for consistency (e.g., “psb28” was changed to “psbW”).

### Organelle genome co-linear analysis

Plastid genome architecture (i.e., arrangements of conserved regions) was investigated using Mauve V.2.3.1 [23] with the progessiveMauve algorithm and default settings with the first anchor position in *rpl9* gene for all plastid genomes. The ''backbone'' file produced by Mauve was used to compare synteny among the taxa using the R library genoPlotR [24]. Brown algal plastid genome distances were calculated using UniMoG [25]. For further analysis, only the double cut and join (DCJ) and Hannenhalli and Pevzner (HP) models were used, as their results were exactly identical to the restricted DJC and the inversion and translocation models, respectively.

Preliminary co-linear analysis conducted for 33 brown mitochondrial genomes did not reveal major rearrangements. This allowed us to use a BLAST Ring Image Generator (BRIG) map [26] to study full-length mitochondrial genome alignments. BRIG was run with standard blastn settings and identity thresholds set at 70% and 50%.

### Phylogenetic analysis and gene evolutionary rates

The thirty-five mitochondrial protein-coding genes common to all brown algae in our study were used for the phylogenetic analysis. They were the *atp6*, *atp8*, *atp9*, *cob*, *cox1*, *cox2*, *cox3*, *nad1*, *nad2*, *nad3*, *nad4*, *nad4L*, *nad5*, *nad6*, *nad7*, *nad9*, *nad11*, *rpl2*, *rpl5*, *rpl6*, *rpl14*, *rpl16*, *rpl31*, *rps2*, *rps3*, *rps4*, *rps7*, *rps8*, *rps10*, *rps11*, *rps12*, *rps13*, *rps14*, *rps19* and *tatC*. For each of them the DNA sequences were aligned at the amino acid level using MAFFT version 7 [27] in combination with TranslatorX [28] and the alignment was subsequently refined manually using Se-Al version 2.0a11 (http://tree.bio.ed.ac.uk/software/seal/). The thirty-five mitochondrial protein-coding gene alignments were concatenated into one dataset using SequenceMatrix 1.7.6 [29] where each gene represented a partition. Maximum likelihood reconstruction was conducted with IQ-Tree v1.3.0 [30] with an independent GTR substitution model with a 4-class gamma distributed rate heterogeneity (G) for each partition of the concatenated data. Branch supports were obtained with the ultrafast bootstrap (UFBoot) implemented in IQ-Tree [31].

Evolutionary rates were estimated for 170 genes common to all phaeophycean taxa (i.e., 35 genes from the mitochondrial genome and 135 genes from the plastid genome). They were individually aligned following the same strategy as for the phylogenetic analysis (see above). Pairwise nonsynonymous substitution rates (dN) of all 170 proteins were estimated using the R library seqinr [32]. The interquartile range (IQR) of the pairwise dN estimates of each gene and the Pearson’s product moment correlation between the IQR and the median dN of each gene were calculated with a newly designed R script. Single gene trees of the 170 genes common to all phaeophycean taxa were reconstructed following the method used for the concatenated tree. Tree topologies of single genes were compared to the topology of the concatenated trees for the mitochondrial and plastid genes. Comparisons were performed using the R library PhySortR [33] with a minimum support value threshold of 50%.

### Protein structure prediction and analysis of the *cox2* gene

The predictions of secondary structure of the *cox2* protein of *Coccophora langsdorfii* were conducted using the I-TASSER suite [34, 35] implemented on the I-TASSER server (http://zhanglab.ccmb.med.umich.edu/I-TASSER/) [36]. Three independent predictions were performed: (1) prediction of the N-terminal part of the *cox2* gene (i.e. the sequence before the insertion), (2) prediction of the C-terminal part of the *cox2* gene (i.e. the sequence after the insertion) and (3) prediction of the full *cox2* sequence. For the first two predictions I-TASSER was run with default settings. For the third prediction, the I-TASSER algorithm was constrained by using the PDB structure of the cytochrome *c* oxidase from *Thermus thermophilus* (PDB ID: 3S8F) and excluding PDB structures (PDB IDs: 5FVM; 5IJO; 3MLI; 3HB3; 1QGR; 4JSP and 3W3T) and their homologous templates with a cutoff of 70% homology.

### Molecular markers for population study

Using the mtDNA BRIG map (S3 Fig), PCR primers were designed with the dedicated tool implemented in Geneious Pro v7 [18]. Primers were further tested for potential dimers using AmplifX v1.7.0 (http://crn2m.univ-mrs.fr/pub/amplifx-dist). The new primer set used in this study was atp6-cox3_F (TAA CTA TTT ACT GGT GGG GGT) and atp6-cox3_R (GTA ATC ATT GCG TTA GTT ATC G). PCR amplifications were conducted as described in [37] on two individuals (Gonghyunjin-2, -3) collected on the same day and in the same population as the individual used for genome sequencing (Gonghyunjin-1), and on one individual collected on 25 May 2011 at Jangsa, Gangwon-do, South Korea (Jangsa-1). Sequences were aligned manually in Se-Al version 2.0a11 (http://tree.bio.ed.ac.uk/software/seal/).

## Results and discussion

### The plastid genome of *Coccophora langsdorfii*

#### Description and gene content

The plastid genome (ptDNA) of *Coccophora langsdorfii* is a 124,450 bp circular molecule (GenBank accession number KU255795). This genome size is within the range of others reported for species in the Fucales (124,068–124,986 bp) but is smaller than those of the Ectocarpales (139,954 bp) and Laminariales species (129,947–130,584 bp) (Table 1). The average GC-content of *C*. *langsdorfii* ptDNA is 29.8%, which falls in the range of reported phaeophycean ptDNAs (28.9–31.1%). The phaeophycean plastid genome has a typical quadripartite organization that is divided into large (LSC) and small (SSC) single-copy regions by two 5,423 bp inverted repeats (IRs) (S1 Fig). The sizes of the different components of brown algal ptDNAs are comparable, with the exception of *E*. *siliculosus*, which has significantly larger IRs (8,615 bp) than those of the other species (5,015–5,446 bp). Without plastid genome sequences from earlier divergent lineages (e.g., Ishigeaceae, Desmarestiales or Dictyotales), it is hard to determine if the IRs in the Ectocarpales have expanded or if IRs have been reduced independently in the Fucales and Laminariales.

**Table 1:** Summary of the general features of the complete plastid genomes of the class Phaeophyceae.

Order	Species	Genome size (bp)	Genome composition (bp) SSC/LSC/IR	Overall GC content (%)	Gene number rRNA/tRNA/CDS/Total	Introns	GenBank accession	References
Fucales	*Coccophora langsdorfii*	124,450	39,858 / 73,747 / 5,423	29.8	6 / 28 / 137 / **173**	1	NC_032288	This study
	*Fucus vesiculosus*	124,986	39,959 / 74,287 / 5,370	28.9	6 / 28 / 137 / **173**	1	NC_016735	[43]
	*Sargassum horneri*	124,068	39,885 / 73,311 / 5,436	30.6	6 / 28 /137 / **173**	2	NC_029856	[44]
	*Sargassum thunbergii*	124,592	40,032 / 73,668 / 5,446	30.4	6 / 28 / 137 / **173**	1	NC_029134	[45]
Laminariales	*Costaria costata*	129,947	42,622 / 76,507 / 5,409	30.9	6 / 28 / 139 / **173**	1	NC_028502	[46]
	*Saccharina japonica*	130,584	43,174 / 77,378 / 5,015	31.1	6 / 29 / 139 / **174**	1	NC_018523	[47]
	*Undaria pinnatifida*	130,383	42,977 / 76,598 / 5,404	30.6	6 / 29 / 139 / **174**	1	NC_028503	[48]
Ectocarpales	*Ectocarpus siliculosus*	139,954	42,714 / 80,010 / 8,615	30.7	6 / 31 / 148 / **185**	1	NC_013498	[43]

*Coccophora langsdorfii* ptDNA contains 139 protein-coding genes (including 16 hypothetical proteins and two *orf*s), 28 tRNAs and 6 rRNAs. The plastid gene set of *C*. *langsdorfii* is identical to that of other algae in the Fucales that have been studied (*F*. *vesiculosus*, *Sar*. *horneri* and *Sar*. *thunbergii*). This gene set is largely shared among phaeophyceaen taxa. The conservation of gene contents appears to be common to most plastid genomes. For instance, the ptDNA in land plants and florideophycean red algae have been shown to be remarkably conserved [38–40].

**Fig 1:**
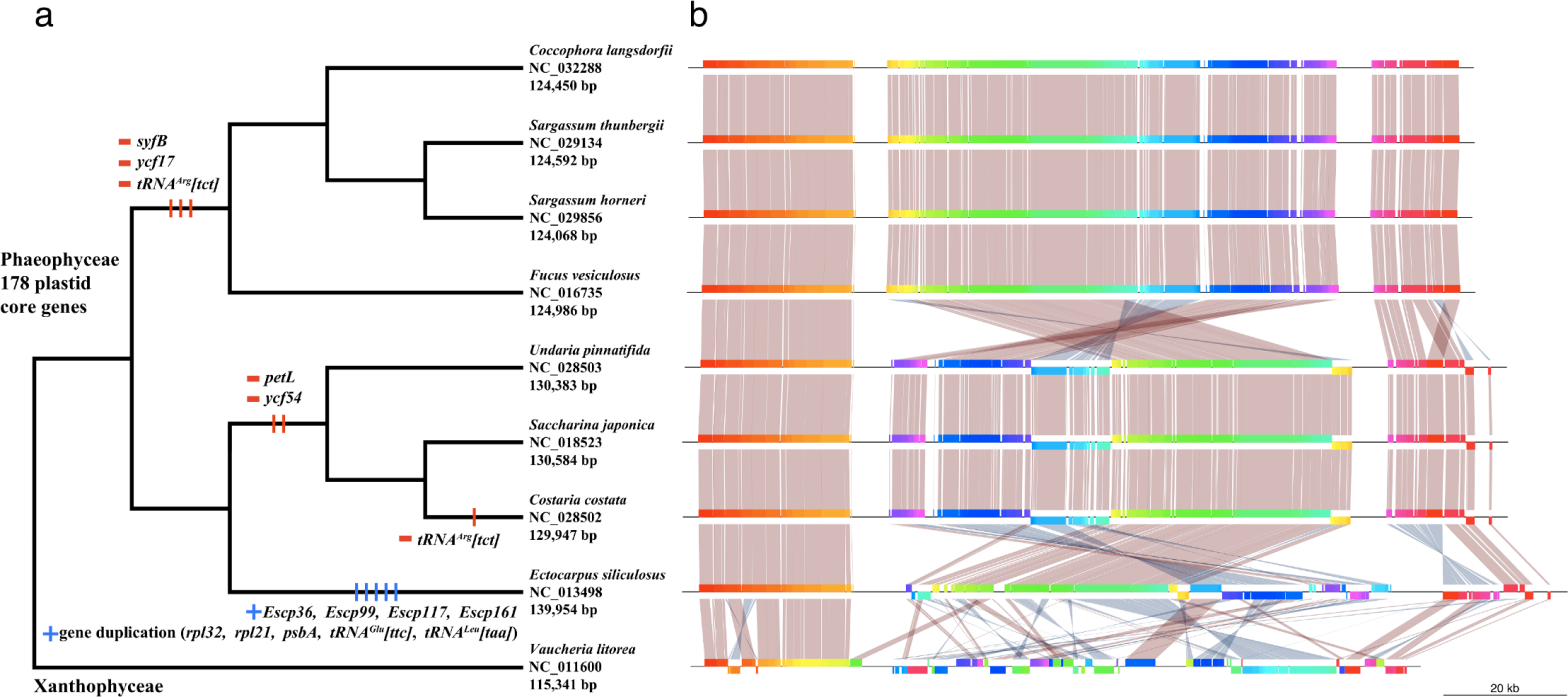
Evolutionary history of the plastid genome in the class Phaeophyceae. The tree topology is based on the current view of the phylogeny of the class Phaeophyceae [41, 42]. Boxes near the nodes represent evolutionary events that occurred along the Phaeophyceae plastid tree. Symbol of minus (–) indicates loss event, whereas symbol of plus (+) indicates gain event. Full alignments were performed using progressiveMauve algorithm [23] and visualized with the genoPlotR library [24]. Colored boxes indicate Locally Collinear Blocks (LCBs) that represent homologous gene clusters.

On the basis of all published brown algal ptDNA datasets, we determined a 178 core gene set that is putatively ancestral and mapped the specific gene loss or gain of lineages in the brown algal phylogenetic tree (Fig 1A). The fucalean ptDNAs lack *syfB*, *ycf17* and *tRNA*^*Arg*^*[tct]* genes whereas the Laminariales lack *petL* and *ycf54* (*Cos*. *costata* also lacks *tRNA*^*Arg*^*[tct]*). The ptDNA of *E*. *siliculosus* is distinguished by four additional *orfs* (*Escp36*, *Escp99*, *Escp117* and *Escp161*) and five duplicated genes found in the IRs (*rpl21*, *rpl32*, *psbA*, *tRNA*^*Leu*^*[taa]* and *tRNA*^*Glu*^*[ttc]*). Because genes that are missing in the Fucales and Laminariales are found in the ptDNAs of other stramenopile lineages (e.g., Xanthophyceae, Bacillariophyceae), it is highly likely that the ancestral plastid genome of brown algae contains these genes. On the other hand, the four *orfs* specific to *E*. *siliculosus* showed no similarity with other brown algal sequences in our BLAST search (data not shown), suggesting independent gene gains in this lineage. Because ptDNA of *E*. *siliculosus* retains all 178 core genes plus nine additional genes, the genome of *E*. *siliculosus* represents the closest approximation to the ancestral plastid genome of the Phaeophyceae.

#### Codon usage

The vast majority of the protein coding genes (137 genes, 98.5%) present in the plastid genome of *C*. *langsdorfii* uses ATG as the start codon, as reported in most of the protein coding genes of the Phaeophyceae [45, 48] (Table 2). Only *rpl3* and *rps8* genes of *C*. *langsdorfii* use ATT as alternate start codon. Interestingly, *rpl3* and *rps8* in other brown algal species have the alternative start codon GTG rather than ATT. This start codon diversity indicates relaxed selection on the *rpl3* and *rps8* genes.

**Table 2:** Codon usage comparison of the complete plastid genomes of the class Phaeophyceae.

Order	Species	Start Codon					Stop Codon		
		ATG	GTG	TTG	ATA	ATT	TAA	TAG	TGA
Fucales	*Coccophora langsdorfii*	137	-	-	-	2 (*rps8*, *rpl3*)	107	26	6
	*Fucus vesiculosus*	136	2 (*psbF*, *rpl3*)	-	-	1 (*orf76*)	113	17	9
	*Sargassum horneri*	138	1 (psbF)	-	-	-	111	25	3
	*Sargassum thunbergii*	136	2 (*psbF*, *rpl3*)	1 (*rps18*)	-	-	108	25	6
Laminariales	*Costaria costata*	136	2 (*psbF*, *rps8*)	-	-	1 (*atpA*)	118	20	1
	*Saccharina japonica*	137	2 (*psbF*, *rps8*)	-	-	-	114	24	1
	*Undaria pinnatifida*	135	2 (*psbF*, *rps8*)	-	1 (*ycf66*)	1 (*atpA*)	116	21	2
Ectocarpales	*Ectocarpus siliculosus*	144	3 (*rps8*, *rpl3*, *rbcR*)	1 (*Escp99*)	-	-	129	14	5

Translational termination codons in the *C*. *langsdorfii* ptDNA are TAA (107 genes, 77%), TAG, (26 genes, 19%) and TGA (6 genes, 4%), similar to codon usages in other phaeophycean species (see Table 2). The stop codon TAG is used for an average of 16.5% of the genes in the Fucales and 15.5% in the Laminariales, whereas it is used in only 9.5% of the genes of the *E*. *siliculosus*. The stop codon TGA is used in only one or two genes in the Laminariales (less than 1% of the total encoded protein coding genes) whereas it is used more broadly in the Fucales and Ectocarpales (average 5% and 3.3% of the total encoded protein-coding genes, respectively).

#### Co-linear genome rearrangement analysis

To investigate rearrangement events in the ptDNAs of brown algal species, we performed a co-linear analysis of all available phaeophyceaen species with the xanthophycean *Vaucheria litorea* (NC_011600) as outgroup (Fig 1). Syntenies of the four Fucales ptDNAs (*C*. *langsdorfii*, *F*. *vesiculosus*, *Sar*. *horneri* and *Sar*. *thunbergii*) are identical. Similarly, three Laminariales species (*Cos*. *costata*, *Sac*. *japonica* and *U*. *pinnatifida*) show identical genome architecture. This indicates that no recombination events occurred after the divergence of the phaeophycean orders. The genome architecture is therefore fixed at the ordinal level. To test this ordinal level of conservation in genome architecture, additional plastid genome data are needed.

In contrast, numerous recombinations are observed in the ptDNAs among different orders (Fig 1B). To assess the number of recombination events, we calculated genome distances between different taxa. The DJC and HP distances were used to represent the number of rearrangement steps necessary to ''reconstruct'' one genome from another. Interestingly, the distances between the genome pairs are not correlated with their phylogenetic relationships. The genome alignment of *E*. *siliculosus* and three Laminariales species (*Cos*. *costata*, *Sac*. *japonica* and *U*. *pinnatifida*) shows a DJC distance of 50 and a HP distance of 52. The Fucales diverged from an ancestor of the Ectocarpales and Laminariales [41] (Fig 1A). However, the DJC and HP distances between the phylogenetically distantly related Fucales (*C*. *coccophora*, *F*. *vesiculosus*, *Sar*. *horneri* and *Sar*. *thunbergii*) and Laminariales are 32 and 34, respectively, while the DJC distance is 58 and HP distance is 59 between *E*. *siliculosus* and the three Fucales species. Thus, architectural rearrangements in ptDNA likely occurred, independent of phylogenetic relationship, in the ancestors of each order. Additional plastid genome sequences from diverse lineages of the brown algae are necessary to fill the gaps in our limited understanding of the evolution of the plastid genome in the Phaeophyceae.

### The mitochondrial genome of *Coccophora langsdorfii*

#### Description and comparison

**Fig 2:**
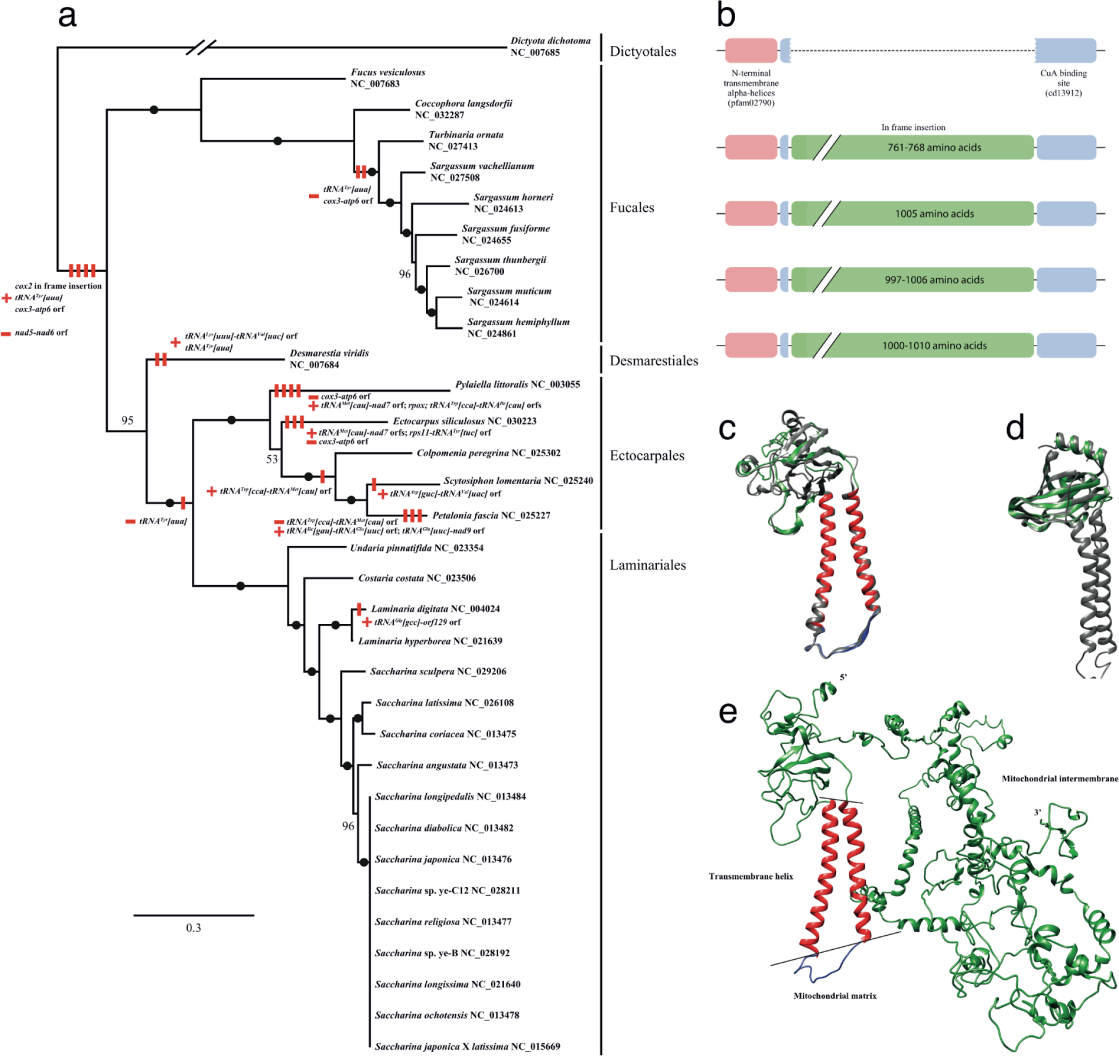
Evolutionary history of the mitochondrial genome in the class Phaeophyceae. The tree was reconstructed with a 35 gene concatenation. Black dots on the branches represent fully supported nodes (100% Maximum Likelihood Support). Bootstrap support values of non-supported nodes are shown near the nodes. Boxes near the nodes represent evolutionary events that occurred along the Phaeophyceae mitochondria tree. Symbol of minus (–) indicates loss event, whereas symbol of plus (+) indicates gain event. Schematic representation of the *cox2* gene in the class Phaeophyceae. The two transmembrane helices are colored in red; the CU_A_ center in blue and the in-frame insert in green.

The mitochondrial genome (mtDNA) of *C*. *langsdorfii* is a circular molecule of 35,660 bp (GenBank accession number KU255794) (S2 Fig). Compared to other fucalean mtDNAs, it is longer than those of six *Sargassum* species and *Turbinaria ornata* but smaller than *F*. *vesiculosus* (Table 3). The overall GC-content is 36.44% and is comparable to that of other fucalean species (i.e., 34.45–37.53%). Non-coding sequences (1,640 bp) represent 4.6% of the *C*. *langsdorfii* mtDNA. This percentage is within the range of that observed in other fucalean species (4.29–5.62%). The mtDNA of *C*. *langsdorfii* contains 38 protein-coding genes (including 3 conserved *orfs*), three rRNAs (*rnl*, *rns* and *rrns*), and 25 tRNAs. All protein-coding and rRNA genes are also found in other fucalean mtDNAs. However, *tRNA*^*Tyr*^*[aua]* genes located between the *tRNA-Ala[ugc]* and *rps10* in *F*. *vesiculosus* and *C*. *langsdorfii* are absent in the six *Sargassum* species and *T*. *ornata*. However, *orf379*, which is also present in *F*. *vesiculosus* and *T*. *ornata*, is present in *C*. *langsdorfii*, indicating that the loss of the *tRNA*^*Tyr*^*[aua]* gene occurred in the common ancestor of the Sargassaceae (including *Coccophora*, *Turbinaria*, and *Sargassum*), whereas *orf379* was lost in the common ancestor of the genus *Sargassum* (Fig 2A).

**Table 3:** Summary of the general features of the complete mitochondrial genomes of the class Phaeophyceae.

Order	Species	Genome size (bp)	Gene number rRNA/tRNA/CDS/Total	Overall GC content (%)	Intergenic spacer (bp)	GenBank accession	References
Fucales	*Coccophora langsdorfii*	35,660	3 / 25 / 35 / **66**	36.4	1,640	NC_032287	This study
	*Fucus vesiculosus*	36,392	3 / 26 / 35 / **67**	34.5	2,045	NC_007683	[49]
	*Turbinaria ornata*	34,981	3 / 25 / 35 / **65**	35.8	1,872	NC_027413	[50]
	*Sargassum fusiforme*	34,696	3 / 25 / 35 / **65**	36.5	1,572	NC_024655	[51]
	*Sargassum hemiphyllum*	34,686	3 / 25 / 35 / **65**	36.6	1,597	NC_024861	[52]
	*Sargassum horneri*	34,606	3 / 25 / 35 / **65**	36.2	1,485	NC_024613	[53]
	*Sargassum muticum*	34,720	3 / 25 / 35 / **65**	36.6	1,614	NC_024614	[54]
	*Sargassum thunbergii*	34,748	3 / 25 / 35 / **65**	36.6	1,600	NC_026700	[55]
	*Sargassum vachellianum*	34,877	3 / 25 / 35 / **65**	36.2	1,629	NC_027508	[56]
Desmarestiales	*Desmarestia viridis*	39,049	3 / 26 / 35 / **68**	36.6	2,366	NC_007684	[49]
Laminariales	*Costaria costata*	37,461	3 / 25 / 35 / **66**	34.9	2,285	NC_023506	[57]
	*Laminaria digitata*	38,007	3 / 24 / 35 / **67**	35.1	2,440	NC_004024	[58]
	*Laminaria hyperborea*	37,976	3 / 23 / 35 / **64**	35.3	2,419	NC_021639	[59]
	*Saccharina angustata*	37,605	3 / 25 / 35 / **66**	35.2	2,328	NC_013473	[60]
	*Saccharina coriacea*	37,500	3 / 25 / 35 / **66**	35.3	2,273	NC_013475	[60]
	*Saccharina japonica*	37,657	3 / 25 / 35 / **66**	35.3	2,444	NC_013476	[60]
	*Saccharina japonica* var. *diabolica*	37,657	3 / 25 / 35 / **66**	35.3	2,412	NC_013482	[60]
	*Saccharina japonica* var. *ochotensis*	37,656	3 / 25 / 35 / **66**	35.3	2,349	NC_013478	[60]
	*Saccharina japonica* var. *religiosa*	37,657	3 / 25 / 35 / **66**	35.3	2,368	NC_013477	[60]
	*Saccharina japonica* x *latissima*	37,638	3 / 25 / 35 / **66**	35.3	2,349	NC_015669	[61]
	*Saccharina latissima*	37,659	3 / 23 / 35 / **65**	35.4	2,442	NC_026108	[62]
	*Saccharina longipedalis*	37,657	4 / 25 / 35 / **66**	35.3	2,368	NC_013484	[60]
	*Saccharina longissima*	37,628	3 / 24 / 35 / **65**	35.3	2,416	NC_021640	[59]
	*Saccharina sculpera*	37,627	2 / 24 / 35 / **64**	35.2	2,531	NC_029206	Direct submission
	*Saccharina* sp. ye-B	37,657	3 / 24 / 35 / **65**	35.3	2,349	NC_028192	Direct submission
	*Saccharina* sp. ye-C12	37,654	3 / 24 / 35 / **65**	35.3	2,349	NC_028211	Direct submission
	*Undaria pinnatifida*	37,402	3 / 24 / 35 / **65**	32.5	2,181	NC_023354	[63]
Ectocarpales	*Colpomenia peregrina*	36,025	3 / 25 / 35 / **66**	31.9	1,499	NC_025302	[64]
	*Ectocarpus siliculosus*	37,189	3 / 25 / 35 / **68**	33.5	2,359	FP885846	[65]
	*Petalonia fascia*	38,053	3 / 25 / 35 / **68**	33.6	2,475	NC_025227	[66]
	*Pylaiella littoralis*	58,507	3 / 24 / 36 / **79**	37.9	3,983	NC_003055	[67]
	*Scytosiphon lomentaria*	36,918	3 / 25 / 35 / **67**	34.1	2,221	NC_025240	[68]
Dictyotales	*Dictyota dichotoma*	31,617	3 / 25 / 35 / **66**	36.5	1,015	NC_007685	[49]

#### Codon usage

Of the 38 mitochondrial protein coding genes in *C*. *langsdorfii*, 37 use ATG as a start codon, similar to other brown algae (Table 4). The *orf379* of *C*. *langsdorfii* and *F*. *vesiculosus* has the alternative start codons ATA or GTG, respectively. Interestingly, in all the Ectocarpales sequenced, the *nad11* gene uses various alternative start codons but never ATG. For example, the *nad11* gene in *Colpomenia peregrina*, *Petalonia fascia* and *Pylaiella littoralis*, uses TTG [64, 66, 67], in *Scytosiphon lomentaria* GTG [68], and in *E*. *siliculosus*, CTG (Table 4). As in the plastid genomes, this suggests that alternative start codon usage is gene-specific and could be related to the transcription efficiency of each gene.

**Table 4:** Codon usage comparison for the complete mitochondrial genomes of the class Phaeophyceae.

Order	Species	Start Codon					Stop Codon		
		ATG	ATA	GTG	TTG	CTG	TAA	TAG	TGA
Fucales	*Coccophora langsdorfii*	37	1 (*orf379*)	-	-	-	26	6	6
	*Fucus vesiculosus*	37	-	1 (*orf379*)	-	-	31	5	2
	*Turbinaria ornata*	37	-	-	-	-	26	7	4
	*Sargassum fusiforme*	37	-	-	-	-	25	8	4
	*Sargassum hemiphyllum*	37	-	-	-	-	25	6	6
	*Sargassum horneri*	37	-	-	-	-	24	6	7
	*Sargassum muticum*	37	-	-	-	-	26	5	6
	*Sargassum thunbergii*	37	-	-	-	-	28	5	4
	*Sargassum vachellianum*	37	-	-	-	-	26	6	5
Desmarestiales	*Desmarestia viridis*	38	-	-	1 (*orf211*)	-	31	6	2
Laminariales	*Costaria costata*	38	-	-	-	-	27	7	4
	*Laminaria digitata*	38	-	1 (*orf157*)	-	-	29	6	4
	*Laminaria hyperborea*	38	-	-	-	-	28	6	4
	*Saccharina angustata*	38	-	-	-	-	29	5	4
	*Saccharina coriacea*	38	-	-	-	-	27	7	4
	*Saccharina japonica*	38	-	-	-	-	26	8	4
	*Saccharina japonica* var. *diabolica*	38	-	-	-	-	26	8	4
	*Saccharina japonica* var. *ochotensis*	38	-	-	-	-	26	8	4
	*Saccharina japonica* var. *religiosa*	38	-	-	-	-	26	8	4
	*Saccharina japonica* x *latissima*	38	-	-	-	-	26	8	4
	*Saccharina latissima*	38	-	-	-	-	28	6	4
	*Saccharina longipedalis*	38	-	-	-	-	26	8	4
	*Saccharina longissima*	38	-	-	-	-	26	8	4
	*Saccharina sculerpa*	38	-	-	-	-	25	8	5
	*Saccharina* sp. ye-B	38	-	-	-	-	26	8	4
	*Saccharina* sp. ye-C12	38	-	-	-	-	26	8	4
	*Undaria pinnatifida*	38	-	-	-	-	30	5	3
Ectocarpales	*Colpomenia peregrina*	37	-	-	1 (*nad11*)	-	31	4	3
	*Ectocarpus siliculosus*	39	-	-	-	1 (*nad11*)	27	8	5
	*Petalonia fascia*	39	-	-	1 (*nad11*)	-	29	8	3
	*Pylaiella littoralis*	51	-	-	1 (*nad11*)	-	38	7	7
	*Scytosiphon lomentaria*	38	-	1 (*nad11*)	-	-	35	2	2
Dictyotales	*Dictyota dichotoma*	36	-	1 (*rps14*)	1 (*orf37*)	-	24	6	8

#### Genome rearrangement analysis

Using a BRIG map [26], we compared the identity of *C*. *langsdorfii* mtDNA to 30 brown algal mtDNAs. In general, gene orders among brown algal mtDNAs are highly conserved with only minor rearrangements. They are mostly insertion/deletion of *orf*s and/or *tRNA* genes, but the gene order of protein-coding genes (*atp8*, *atp9*, *rpl31* and *rps10*) in *D*. *dichotoma* differs from those of other species [49]. In *C*. *langsdorfii* mtDNA, the genome has a 50 to 70% identity to the genomes of other brown algal species (S3 Fig), and the *rns* and *rnl* genes have an identity with other species higher than 70%.

Seven regions show higher genetic divergences than the rest of genomic regions: the *tRNA*^*Pro*^*[ugg]-rnl* spacer, the *rnl-tRNA*^*Lys*^*[uuu]* spacer, the *cox3-atp6* spacer (containing various *orf*s), the *cob-cox2* spacer, the *cox2* gene, the *nad6* gene, and the region between the *rps14* and *rns* genes (S3 Fig). In *Sargassum* species, the *cox3-atp6* spacer was found to be the most variable region in terms of both sequence identity and spacer length [55]. This spacer contains the conserved *orf379* that is found only in *F*. *vesiculosus* and *C*. *langsdorfii*. The reduced size (1,140 bp in *F*. *vesiculosus* compared to 849 bp in *C*. *langsdorfii*) suggests potential pseudogenization in *C*. *langsdorfii*.

#### The cytochrome *c* oxidase subunit 2

The high variability observed in the *cox2* region is due to the presence of a large in-frame insertion found only in the orders of Fucales, Desmarestiales, Laminariales and Ectocarpales (S4 and S5 Figs). Because of this insertion, the length of the *cox2* gene in the Stramenopiles varies from 687–906 bp (without insertion, as in the Dictyotales, Bacillariophyceae, and Oomycota) to 3,129–3,921 bp (with insertion) (S5 Fig). This in-frame insertion shows a high level of conservation among brown algal species (S4 Fig). Furthermore, the insert-containing lineages form a well-supported monophyletic group (Fig 2A). Therefore, it is likely that there was a single in-frame insertion event in the *cox2* gene of the ancestor of these four brown algal orders. Similarly, a large insert was also found in the *cox2* gene of the ciliate genera *Euplotes* (3,051–3,063 bp), *Tetrahymena* (1,812 bp) and *Paramecium* (1,728 bp) [69, 70], which might be derived from a common ancestor [69]. Interestingly, despite its independent origins, the insertion in the *cox2* gene is situated at the same position in brown algae and ciliates. Those inserts separate the two functional domains of the *cox2* subunit: the two N-terminal transmembrane helices and the C-terminal copper A center (CU_A_) (Fig 2B). Furthermore, the *cox2* genes found in alveolates, chlorophycean algae and in one species of Hymenoptera (insects) have been independently split into two subunits corresponding to the N-terminal and C-terminal domains of the canonical *cox2* gene [71–74]. The split of the *cox2* gene occurred at the same location as the insertions in other lineages. All of these events appear to be lineage-specific and independent. Other genes have been reported as split (e.g. *SdhB* in euglenozoans [75], *rpl2* in some angiosperms [76] or *nad1* in ciliates [70]) but it is remarkable that the *cox2* gene is undergoing independent evolutionary events in many different lineages. Furthermore, most of the split mitochondrial genes were subsequently transferred to the nucleus [72–74]. The insert observed in the brown algae might represent an early stage in a similar split and later transfer to the nucleus. However, this is extremely speculative and further analyses, especially regarding expression, are required.

Predictions of the secondary structure of the N-terminal and C-terminal yielded structures comparable to the canonical *cox2* protein (Fig 2C and 2D). Conservation of those domains would suggest that despite the large insert, the brown algal *cox2* gene still encodes a functional *cox2* protein. However, the *cox2* protein is a subunit of the cytochrome *c* oxidase complex and therefore has to be precisely assembled with the *cox1* and *cox3* subunits to form a functional complex [77]. This correct assemblage is the foundation of the entire mitochondrial transport chain [78]. To determine how the insert could affect the assemblage of the cytochrome *c* oxidase complex, we predicted the structure of the *cox2* protein in *C*. *langsdorfii*. The prediction failed to recover a three dimensional organization that would bring the C-terminal CU_A_ center close to the position in which it is normally found (Fig 2E). If the CU_A_ center of the *cox2* subunit is not in the right position, electrons might not be transferred from the *cox2* subunit to the *cox1* subunit. In mammals, a misassembled cytochrome *c* oxidase causes serious pathologies and can be lethal [78]. Yet the highly modified *cox2* subunit in the brown algae has not been eliminated by natural selection, suggesting that the brown algal cytochrome *c* oxidase has an assemblage that differs from those found so far in other organisms. Understanding the structure and assemblage of this unique cytochrome *c* oxidase would contribute considerable insight into the physiology of the brown algae and possibly into their ecological success. This, however, remains to be explored.

### Molecular markers

#### Evolutionary divergence rates of organelle genes

In general, organelle genomes are present in multiple copies in a cell and their genes can be easily amplified and sequenced, making them an important source of molecular markers for phylogenetic studies or barcoding. Despite their advantages, only a handful of organelle genes have been widely used (mainly 16S rRNA, *rbcL*, *psbA*, *cox1*) in phylogenetic studies [41, 79, 80].

To investigate their value as molecular markers, we estimated the evolutionary divergence rate for the genes found in the plastid and mitochondrial genomes of the Phaeophyceae, following the approach developed by Janouškovec *et al*. [39]. Briefly, we used 35 complete mitochondrial and eight complete plastid genomes to calculate pairwise comparisons of nonsynonymous substitutions per site (dN) and their standard deviation (interquantile range, IQR) for a total of 170 genes. These dNs were used to estimate the divergence rate of individual genes, while the IQRs were used to estimate the uniformity of dN rates among species. Genes were ordered by their median dN (S6 Fig).

**Fig 3:**
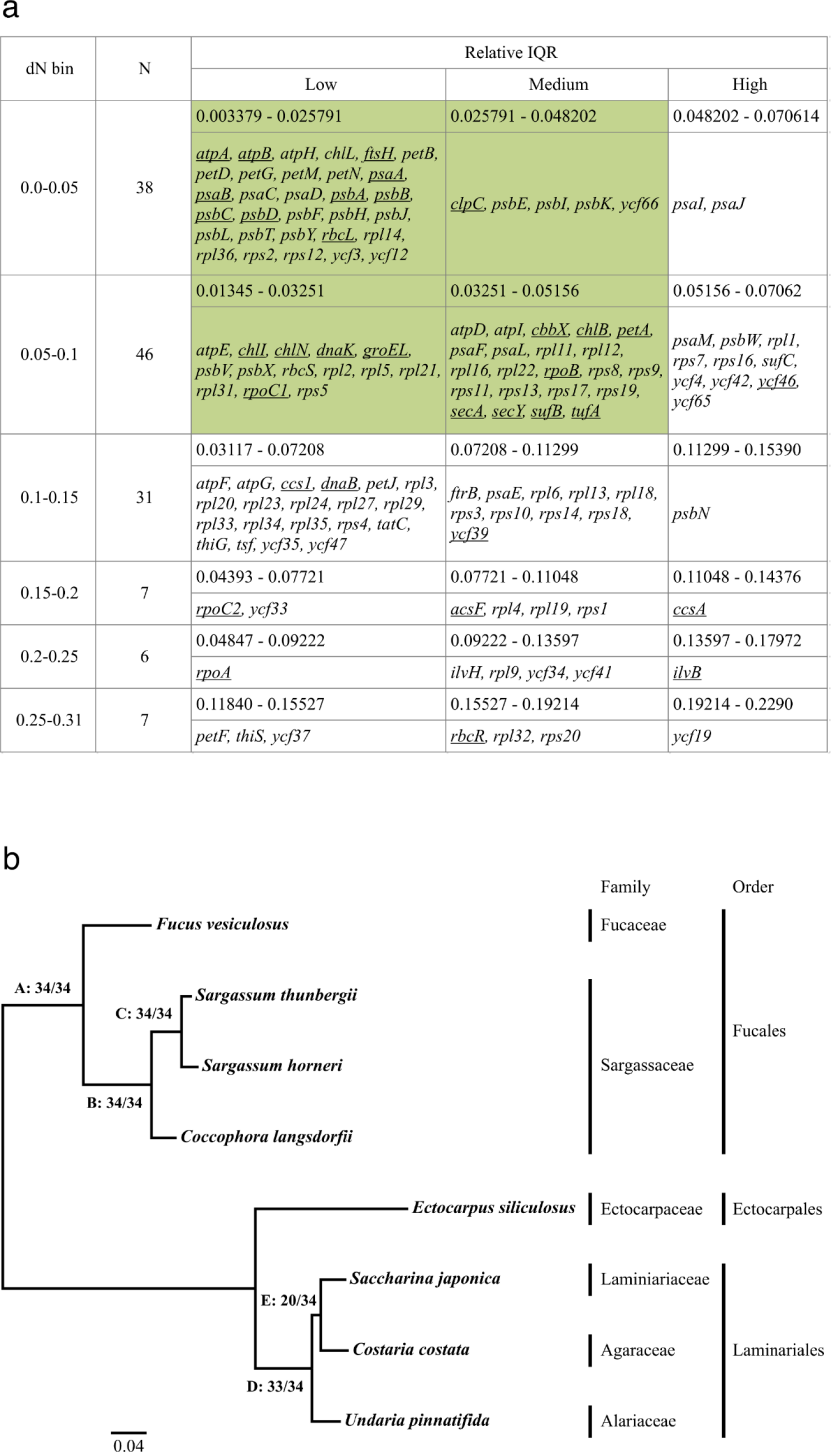
Genetic evolutionary rates of the plastid encoded genes of the Phaeophyceae. (a) Summary of the characteristics of individual genes by dN bin and IQR bin. Bins highlighted are potential candidate genes for phylogenies and discrimination of orders or families. Underlined genes are 900 bp or more length. (b) Maximum likelihood phylogeny reconstructed from the concatenation of genes with a size larger than 300 amino acids. Numbers near the nodes represent the number of single gene trees that support the node.

**Fig 4:**
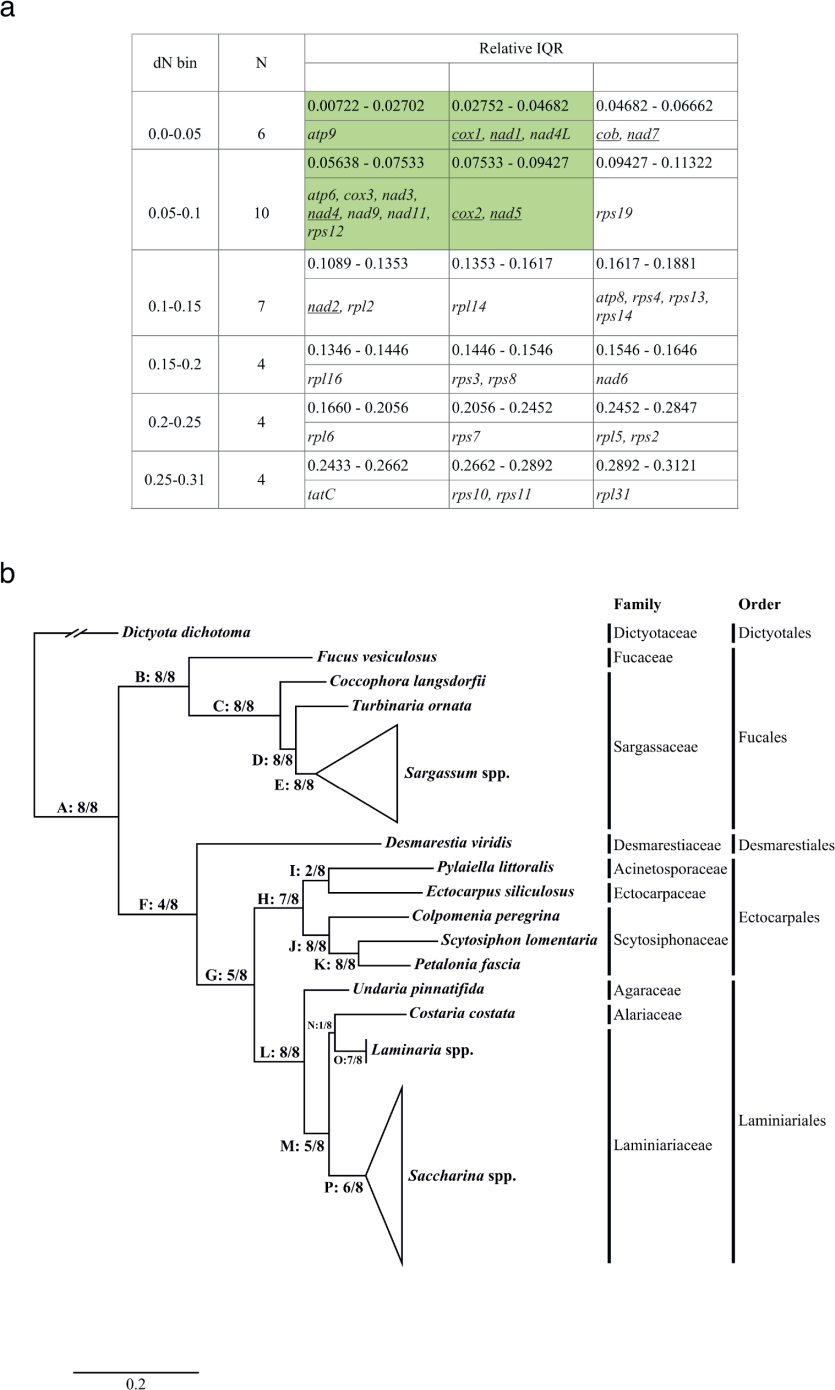
Genetic evolutionary rates of the mitochondrial encoded genes of the Phaeophyceae. (a) Summary of the characteristics of individual genes by dN bin and IQR bin. Bins highlighted are potential candidate genes for phylogenies and discrimination of orders or families. Underlined genes are 900 bp or more. (b) Maximum likelihood phylogeny reconstructed from the concatenation of genes with a size larger than 300 amino acids. Numbers near the nodes represent the number of single gene trees that support the node.

The mitochondrial genes generally showed a higher dN than those of the plastid genes. A similar trend was observed in plastid-bearing species and especially in the species containing red-algal-derived plastids [80]. As in red algal plastid genomes [39], the dNs of the plastid and mitochondrial genes of the brown algae were positively correlated with the IQR (S6 Fig). This means that larger dN correlated with unequal dN distances among species, whereas suitable markers for phylogenies should have close to equal dN rates among species (i.e., low IQR value). Therefore, the 27 genes with the highest IQR may not be appropriate for phylogenetic or studies at the intraspecific level (Figs 3A and 4A). Out of the 143 genes with low to medium IQR, we further excluded genes with lengths less than 900 nucleotides, because shorter genes are not practical for primer design and PCR. More importantly, short sequences may not contain sufficient genetic information to resolve phylogenetic relationships. This analysis resulted in 42 genes in the plastid (34 genes underlined in Fig 3A) and mitochondrial (eight genes underlined in Fig 4A) genomes that we identified as candidates for markers in phylogenetic studies.

#### Phylogenetic analysis using single gene datasets

To test the suitability of these 42 candidate genes for the phylogenetic analysis, we compared the tree topologies of 42 single genes with the concatenated multigene topologies (34 plastid genes and eight mitochondrial genes, see Figs 3B and 4B). The concatenated multigene trees were generally congruent with the published phaeophycean phylogenies [41, 42]. The single gene trees were mostly congruent with the concatenated trees, indicating that information content is consistent over the single genes. This is a valuable finding if those genes are to be used in a concatenated tree for phylogenetic analysis. However, four nodes were poorly supported or not supported by the single gene trees. In the plastid tree, the node E joining *S*. *japonica* and *C*. *costata* was found in only 20 out of 34 single gene trees (Fig 3B). In the remaining trees, *U*. *pinnatifida* was sister to *S*. *japonica*. Internal relationships within kelps (Laminariales) are generally unresolved due to rapid diversification [41, 81].

In the mitochondrial tree, nodes F, I and N were not supported. These nodes occurred at different levels in the tree. The interordinal node F was recovered in four out of eight trees with maximum likelihood bootstrap support (MLB) higher than 50%, and in two other trees with lower MLB support. The interfamilial node I grouping the families of Acinetosporaceae and Ectocarpaceae was recovered in only two out of eight trees. In the remaining six trees, the family Ectocarpaceae was sister to the family Scytosiphonaceae, but this position was strongly supported in only one tree. The node N that grouped the genera *Costaria* and *Laminaria* was not recovered in seven single gene trees. This is not congruent with the latest phylogenetic study of the order Laminariales [82] and could point to a lack of phylogenetic information in our 42 candidate genes and/or be an artifact of the limited number of taxa included in our analysis. As in the plastid trees, this lack of resolution might also be explained by rapid diversification of the order Laminariales [41, 81].

#### Proposed markers for phylogenetic analysis

We propose a set of 29 genes that would be efficient and effective for phylogenetic analysis: the plastid-encoded *atpA*, *atpB*, *cbbX*, *chlB*, *chlI*, *chlN*, *clpC*, *dnaK*, *ftsH*, *groEL*, *petA*, *psaA*, *psaB*, *psbA*, *psbB*, *psbC*, *psbD*, *rbcL*, *rpoB*, *rpoC1*, *secA*, *secY*, *sufB*, *tufA* genes and the mitochondria-encoded *cox1*, *cox2*, *nad1*, *nad4* and *nad5* genes. Although the limited number of species used in our analyses might affect the delimitation of the IQR categories and the topology of trees, all the phaeophycean organelle genomes reported so far contained these genes, so they may assist in resolving problematic brown algal phylogenetic relationships at various levels [41, 82]. Other genes, such as the plastid encoded *acsF*, *rbcR*, *rpoA*, and *rpoC2*, with higher dN values (0.15–0.31) and lengths greater than 900 bp could be used for the study of subspecies and populations, including a study of gene flow and connectivity among populations of *C*. *langsdorfii*.

#### Proposed marker for population genetic structure and conservation studies

Conservationists use molecular markers to determine threats menacing endangered species (e.g., genetic homogeneity or isolation of populations, small effective population size). Indeed, an understanding of the level of genetic diversity among individuals and populations promotes the understanding of population dynamics and the consequences of those threats [9]. Organellar genomes are valuable sources for molecular markers potentially suitable for the analysis of population genetic structure and processes. We mined the mtDNA sequence of *C*. *langsdorfii* in an effort to develop such molecular markers.

Using a BRIG map [26] we identified five regions of genetic diversity higher than the rest of the genome in the fucalean mtDNA (S3 Fig). These were the *tRNA*^*Pro*^*[ugg]-rnl* spacer, the *rnl-tRNA*^*Lys*^*[uuu]* spacer, the *cox3-atp6* spacer, the *cox2* gene, and the region between the *rps14* and *rns* genes These regions were chosen as candidate markers. We removed the *tRNA*^*Pro*^*[ugg]-rnl* spacer and the *rnl-tRNA*^*Lys*^*[uuu]* spacer from consideration because they were too short (48 bp and 160 bp, respectively). We also removed the *cox2* gene and the region between the *rps14* and *rns* genes because they were too long (3,144 bp and 1,433 bp, respectively).

**Fig 5:**
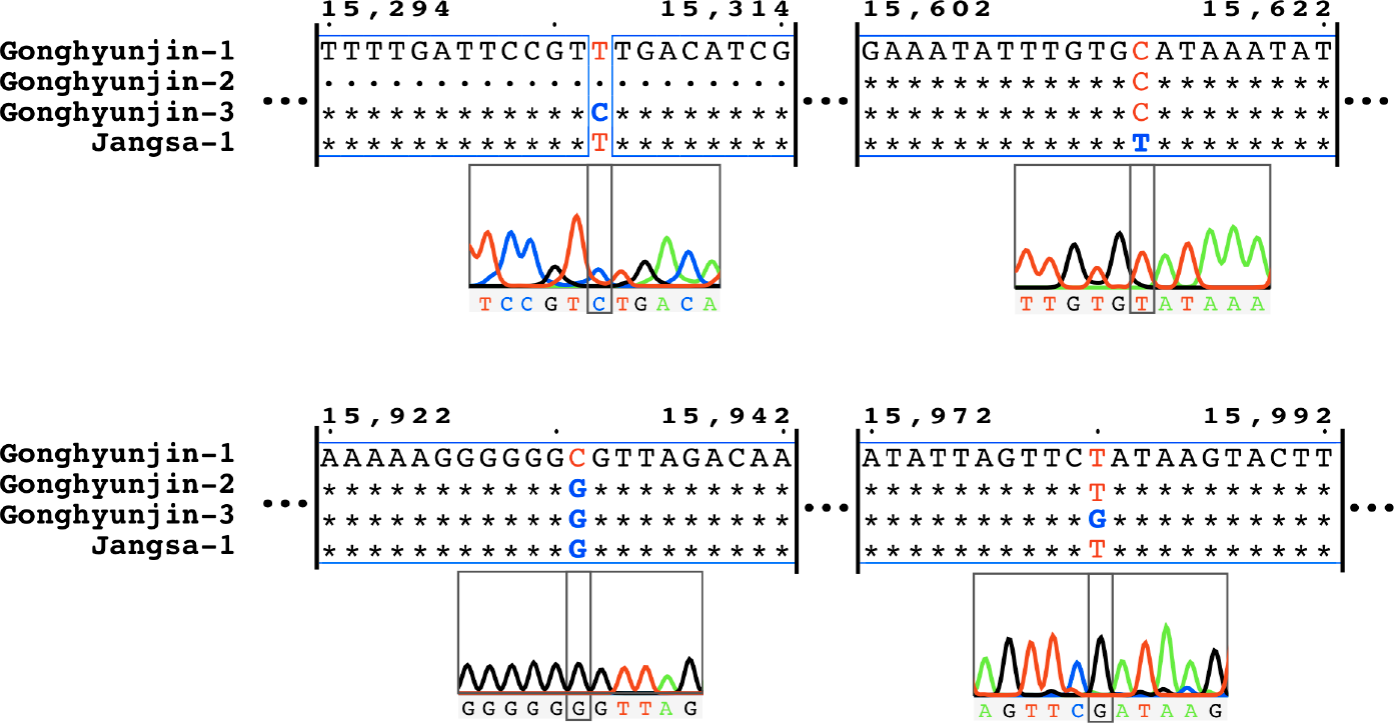
Sequence alignment of the *atp6-cox3* spacer in *Coccophora langsdorfii*. Asterisks represent sites with no polymorphism. Blue frames represent conserved regions. Red characters represent the reference polymorphism and blue bold characters represent individual polymorphism. Chromatograms illustrate the sequencing quality at the polymorphic sites (grey box) and 5 bp up- and downstream.

To test intraspecific diversity in the *atp6-cox3* spacer, we sequenced this region for additional three individuals of *C*. *langsdorfii* belonging to two populations in Gonghyunjin and Jangsa, Korea. We obtained a 970 bp alignment that contained four polymorphic sites (Fig 5). To verify the stability of these polymorphic sites, we conducted independent PCR and Sanger sequencing, using the chromatogram to confirm the quality of the sequencing at those sites. The four sites showed clear single peak in their respective chromatograph, validating these polymorphisms. Interestingly, polymorphisms were identified among individuals belonging to the same population (Gonghyunjin-1, Gonghyunjin-2 Gonghyunjin-3 from Gonghyunjin, Korea). This suggests that the *atp6-cox3* spacer is a strong candidate as a marker to quantify genetic diversity within and between populations of *C*. *langsdorfii*. Our new primer set (atp6-cox3_F and atp6-cox3_R) should be used to conduct a large-scale investigation of the population structure of *C*. *langsdorfii* to establish an efficient conservation strategy for this alga.

## Supporting information

S1 Fig Plastid genome map of *Coccophora langsdorfii*. Annotated genes are color-coded according to their function. Genes on the outside of the circles are transcribed clockwise and those on the inside counter clockwise. Abbreviations: IR, Inverse Repeats; SSC, Small Single-Copy; LSC, Large Single-Copy.
(PDF)

S2 Fig Mitochondrial genome map of *Coccophora langsdorfii*. Annotated genes are color-coded according to their function. Genes on the outside of the circles are transcribed clockwise and those on the inside counter clockwise.
(PDF)

S3 Fig Full length comparison of 31 mitochondrial genomes of the class Phaeophyceae. The innermost ring represents the mitochondrial genome map of *Coccophora langsdorfii*, annotated as in Fig 1. Every colored rings represent BLAST comparisons of a complete mitochondrial genomes against *Coccophora langsdorfii*. From the inside to the outside are represented the Fucales in red (*Sargassum thunbergii*, *Sargassum vachellianum*, *Sargassum fusiforme*, *Sargassum hemiphyllum*, *Sargassum horneri*, *Sargassum muticum*, *Turbinaria ornata*, *Fucus vesiculosus*), the Desmarestiales in blue (*Desmarestia viridis*), the Laminariales in brown (*Undaria pinnatifida*, *Costaria costata*, *Laminaria digitata*, *Laminaria hyperborea*, *Saccharina angustata*, *Saccharina latissima*, *Saccharina coriaceae*, *Saccharina japonica*, *Saccharina* sp. ye-C12, *Saccharina religiosa*, *Saccharnia ochotensis*, *Saccharina japonica*X*latissima*, *Saccharina longissima*, *Saccharina longipedalis*, *Saccharina diabolica*, *Saccharina* sp. ye-B), the Ectocarpales in green (*Colpomenia peregrina*, *Scytosiphon lomentaria*, *Petalonia fascia*, *Pylaiella littoralis*) and the Dictyotales in purple (*Dictyota dichotoma*). Intensity of the ring color denotes the degree of identity whereas gaps represent highly divergent regions.
(PDF)

S4 Fig Aligned sequences of the *cox2* gene in the class Phaeophyceae. White characters in a red box represent strict identity between all sequences. Red characters represent similarity in a group of sequences. Blue frame represent a conserved region. Green highlight represent the in frame insertion found in the Fucales, Desmarestiales, Ectocarpales and Laminariales.
(PDF)

S5 Fig Length in nucleotide of the mitochondrial *cox2* gene in the different lineages of the Stramenopiles. (PDF)

S6 Fig Plot of median nonsynonymous substitution rate (dN) of protein-coding genes encoded in the mitochondrial genome (in red) and the plastid genome (in green) with interquantile range (IQ). (PDF)

S1 Table Blastp summary and unified proposed annotation. *Coccophora langsdorfii* sequences were used as query for a blastp search against other brown algae plastid genomes.
(PDF)

S2 Table Summary of the accordance between the plastid encoded single gene trees topologies and the phylogeny of the Phaeophyceae. TRUE indicates that the node is supported with above 50% Maximum Likelihood Bootstrap value. Underlined genes are more than 300 amino acids.
(PDF)

S3 Table Summary of the accordance between the mitochondria encoded single gene trees topologies and the phylogeny of the Phaeophyceae. TRUE indicates that the node is supported with above 50% Maximum Likelihood Bootstrap value. Underlined genes are more than 300 amino acids.
(PDF)
