# Supplementary material for: Plastid and mitochondrial genomes of Coccophora langsdorfii (Fucales, Phaeophyceae) and the utility of molecular markers
Source: PLoS One. 2017 Nov 2;12(11):e0187104. doi: 10.1371/journal.pone.0187104 (PMC5695614; doi:10.1371/journal.pone.0187104)
Supplement: S1 Fig — Annotated genes are color-coded according to their function. Genes on the outside of the circles are transcribed clockwise and those on the inside counter clockwise. Abbreviations: IR, Inverse Repeats; SSC, Small Single-Copy; LSC, Large Single-Copy. (PDF) [file pone.0187104.s001.pdf]

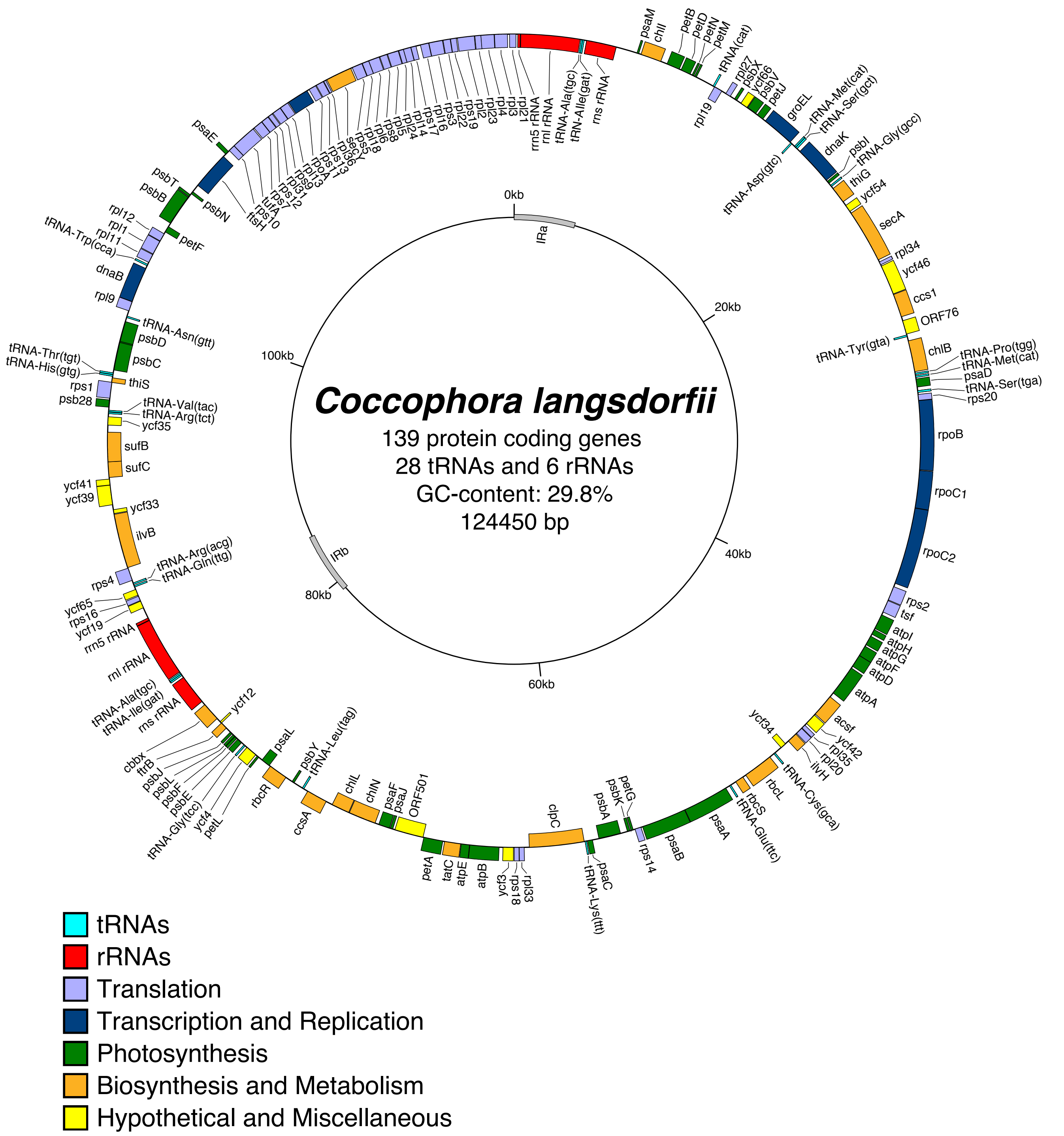

S1 Fig. Plastid genome map of *Coccophora langsdorfii*. Annotated genes are color-coded according to their function. Genes on the outside of the circles are transcribed clockwise and those on the inside counter clockwise. Abbreviations: IR, Inverse Repeats; SSC, Small Single-Copy; LSC, Large Single-Copy.
