# Supplementary material for: Plastid and mitochondrial genomes of Coccophora langsdorfii (Fucales, Phaeophyceae) and the utility of molecular markers
Source: PLoS One. 2017 Nov 2;12(11):e0187104. doi: 10.1371/journal.pone.0187104 (PMC5695614; doi:10.1371/journal.pone.0187104)
Supplement: S3 Fig — The innermost ring represents the mitochondrial genome map of Coccophora langsdorfii, annotated as in Fig 1. Every colored rings represent BLAST comparisons of a complete mitochondrial genomes against Coccophora langsdorfii. From the inside to the outside are represented the Fucales in red (Sargassum thunbergii, Sargassum vachellianum, Sargassum fusiforme, Sargassum hemiphyllum, Sargassum horneri, Sargassum muticum, Turbinaria ornata, Fucus vesiculosus), the Desmarestiales in blue (Desmarestia viridis), the Laminariales in brown (Undaria pinnatifida, Costaria costata, Laminaria digitata, Laminaria hyperborea, Saccharina angustata, Saccharina latissima, Saccharina coriaceae, Saccharina japonica, Saccharina sp. ye-C12, Saccharina religiosa, Saccharnia ochotensis, Saccharina japonicaXlatissima, Saccharina longissima, Saccharina longipedalis, Saccharina diabolica, Saccharina sp. ye-B), the Ectocarpales in green (Colpomenia peregrina, Scytosiphon lomentaria, Petalonia fascia, Pylaiella littoralis) and the Dictyotales in purple (Dictyota dichotoma). Intensity of the ring color denotes the degree of identity whereas gaps represent highly divergent regions. (PDF) [file pone.0187104.s003.pdf]

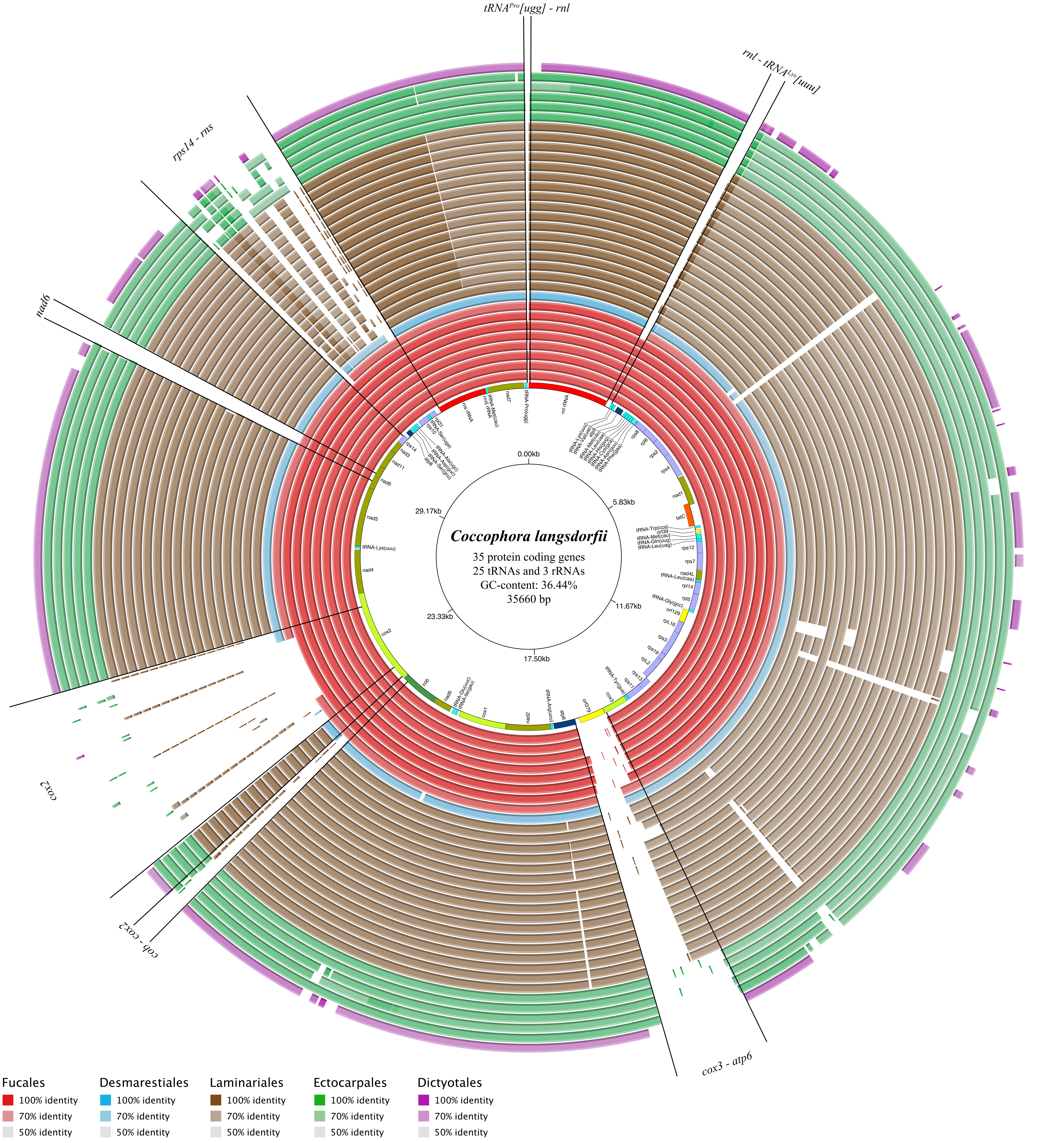

S3 Fig. Full length comparison of 31 mitochondrial genomes of the class Phaeophyceae. The innermost ring represents the mitochondrial genome map of *Coccophora langsdorfii*, annotated as in Fig 1. Every colored rings represent BLAST comparisons of a complete mitochondrial genomes against *Coccophora langsdorfii*. From the inside to the outside are represented the Fucales in red (*Sargassum thunbergii*, *Sargassum vachellianum*, *Sargassum fusiforme*, *Sargassum hemiphyllum*, *Sargassum horneri*, *Sargassum muticum*, *Turbinaria ornata*, *Fucus vesiculosus*), the Desmarestiales in blue (*Desmarestia viridis*), the Laminariales in brown (*Undaria pinnatifida*, *Costaria costata*, *Laminaria digitata*, *Laminaria hyperborea*, *Saccharina angustata*, *Saccharina latissima*, *Saccharina coriaceae*, *Saccharina japonica*, *Saccharina* sp. ye-C12, *Saccharina religiosa*, *Saccharnia ochotensis*, *Saccharina japonica*X*latissima*, *Saccharina longissima*, *Saccharina longipedalis*, *Saccharina diabolica*, *Saccharina* sp. ye-B), the Ectocarpales in green (*Colpomenia peregrina*, *Scytosiphon lomentaria*, *Petalonia fascia*, *Pylaiella littoralis*) and the Dictyotales in purple (*Dictyota dichotoma*). Intensity of the ring color denotes the degree of identity whereas gaps represent highly divergent regions.
