# Supplementary material for: Plastid and mitochondrial genomes of Coccophora langsdorfii (Fucales, Phaeophyceae) and the utility of molecular markers
Source: PLoS One. 2017 Nov 2;12(11):e0187104. doi: 10.1371/journal.pone.0187104 (PMC5695614; doi:10.1371/journal.pone.0187104)
Supplement: S5 Fig — (PDF) [file pone.0187104.s005.pdf]

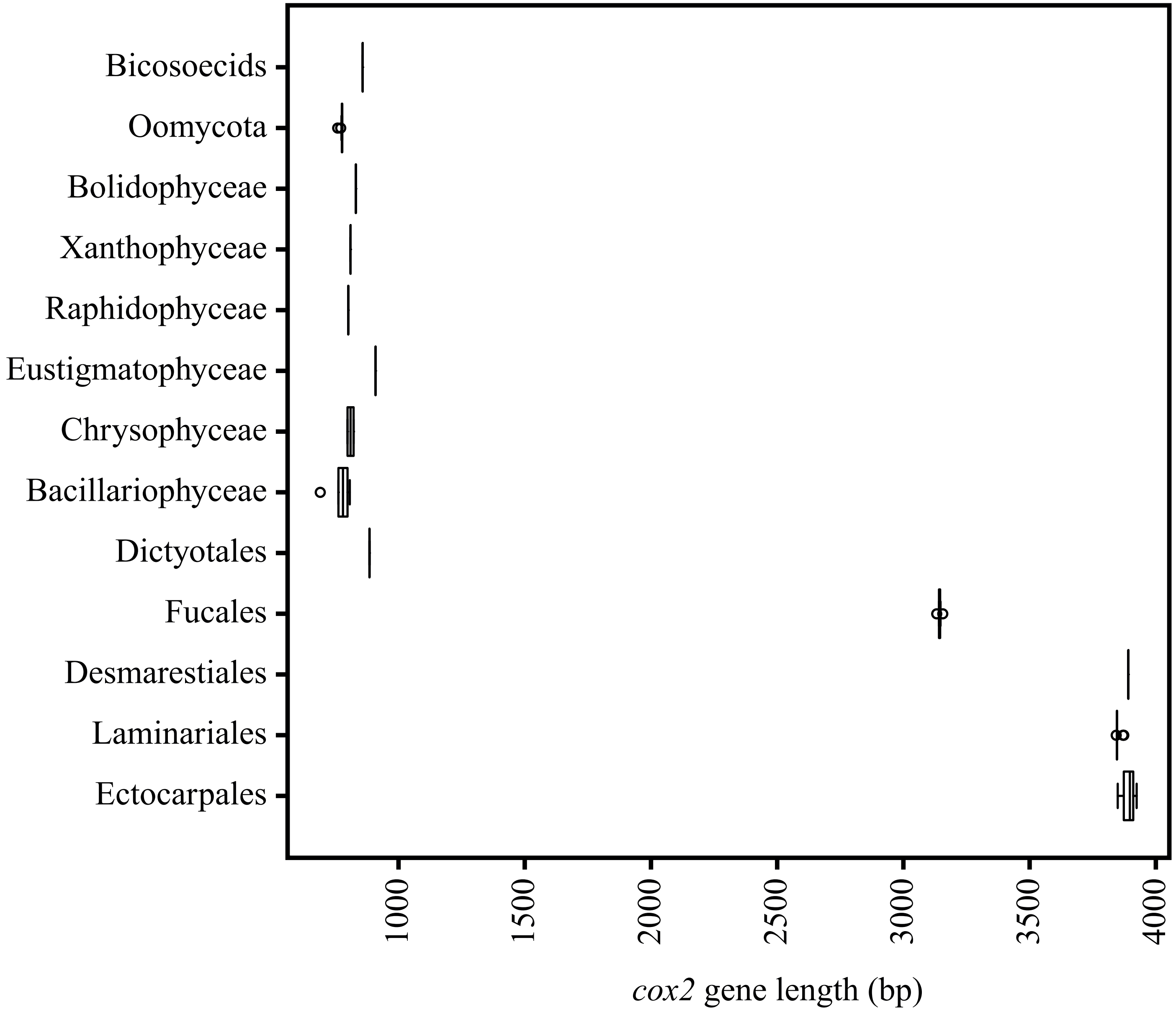

S5 Fig. Length in nucleotide of the mitochondrial cox2 gene in the different lineages of the Stramenopiles.
