# Supplementary material for: Plastid and mitochondrial genomes of Coccophora langsdorfii (Fucales, Phaeophyceae) and the utility of molecular markers
Source: PLoS One. 2017 Nov 2;12(11):e0187104. doi: 10.1371/journal.pone.0187104 (PMC5695614; doi:10.1371/journal.pone.0187104)
Supplement: S1 Table — Coccophora langsdorfii sequences were used as query for a blastp search against other brown algae plastid genomes. (PDF) [file pone.0187104.s007.pdf]

S1 Table. Blastp summary and unified proposed annotation. *Coccophora langsdorfii* sequences were used as query for a blastp search against other brown algae plastid genomes.

Gene	Organism	e-value	Identity	Accession	Annotation	Unified proposed annotation
**thiS**						
	Sargassum thunbergii	2e-40	81%	YP_009227400	thiS	thiS
	Sargassum horneri	6e-40	81%	YP_009243776	thiS	thiS
	Fucus vesiculosus	1e-31	65%	YP_005090112	thiS	thiS
	Saccharina japonica	6e-24	53%	YP_006639134	ycf40	thiS
	Undaria pinnatifida	9e-24	53%	YP_009182614	ycf40	thiS
	Costaria costata	3e-22	52%	YP_009182475	ycf40	thiS
	Ectocarpus siliculosus	53-22	53%	YP_003289161	ycf40	thiS
**sufB**						
	Sargassum thunbergii	0.0	97%	YP_009227396	sufB	sufB
	Sargassum horneri	0.0	97%	YP_009243772	sufB	sufB
	Fucus vesiculosus	0.0	91%	YP_005090108	sufB	sufB
	Saccharina japonica	0.0	88%	YP_006639136	ycf24	sufB
	Undaria pinnatifida	0.0	88%	YP_009182616	ycf24	sufB
	Costaria costata	0.0	88%	YP_009182477	ycf24	sufB
	Ectocarpus siliculosus	0.0	89%	YP_003289159	ycf24	sufB
**sufC**						
	Sargassum thunbergii	6e-177	95%	YP_009227395	sufC	sufC
	Sargassum horneri	4e-177	95%	YP_009243771	sufC	sufC
	Fucus vesiculosus	3e-164	87%	YP_005090107	sufC	sufC
	Saccharina japonica	5e-162	84%	YP_006639137	ycf16	sufC
	Undaria pinnatifida	3e-159	84%	YP_009182617	ycf16	sufC
	Costaria costata	1e-161	84%	YP_009182478	ycf16	sufC
	Ectocarpus	9e-158	84%	YP_009182617	ycf16	sufC

	siliculosus					
**orf501**						
	Sargassum thunbergii	0.0	77%	YP_009227367	orf501	orf501
	Sargassum horneri	0.0	76%	YP_009243743	orf467	orf501
	Fucus vesiculosus	6e-134	47%	YP_005090079	orf501	orf501
	Saccharina japonica	63-88	37%	YP_006639065	LJCPDNA_056	orf501
	Undaria pinnatifida	4e-84	38%	YP_009182545	AT91_gp084	orf501
	Costaria costata	1e-77	39%	YP_009182406	cp74	orf501
	Ectocarpus siliculosus	2e-26	28%	YP_003289263	Escp152	orf501
**orf76**						
	Sargassum thunbergii	3e-146	95%	YP_009227329	orf76	orf76
	Sargassum horneri	2e-146	95%	YP_009243705	orf219	orf76
	Fucus vesiculosus	6e-128	81%	YP_005090041	orf76	orf76
	Saccharina japonica	1e-111	67%	YP_006639029	LJCPDNA_020	orf76
	Undaria pinnatifida	4e-107	65%	YP_009182509	AT91_gp120	orf76
	Costaria costata	2e-112	68%	YP_009182370	cp30	orf76
	Ectocarpus siliculosus	3e-88	52%	YP_003289236	Escp124	orf76
