# Supplementary material for: Plastid and mitochondrial genomes of Coccophora langsdorfii (Fucales, Phaeophyceae) and the utility of molecular markers
Source: PLoS One. 2017 Nov 2;12(11):e0187104. doi: 10.1371/journal.pone.0187104 (PMC5695614; doi:10.1371/journal.pone.0187104)
Supplement: S2 Table — TRUE indicates that the node is supported with above 50% Maximum Likelihood Bootstrap value. Underlined genes are more than 300 amino acids. (PDF) [file pone.0187104.s008.pdf]

**S2 Table. Summary of the accordance between the plastid encoded single gene trees topologies and the phylogeny of the Phaeophyceae.** TRUE indicates that the node is supported with above 50% Maximum Likelihood Bootstrap value. Underlined genes are more than 300 amino acids.

| gene | node A | node B | node C | node D | node E | Total supported nodes |
|---|---|---|---|---|---|---|
| acsF | TRUE | TRUE | TRUE | TRUE | TRUE | 5 |
| atpA | TRUE | TRUE | TRUE | TRUE | TRUE | 5 |
| atpB | TRUE | TRUE | TRUE | TRUE | TRUE | 5 |
| atpD | TRUE | TRUE | TRUE | TRUE | FALSE | 4 |
| atpE | TRUE | TRUE | TRUE | FALSE | FALSE | 3 |
| atpF | TRUE | TRUE | TRUE | TRUE | FALSE | 4 |
| atpG | TRUE | TRUE | TRUE | TRUE | FALSE | 4 |
| atpH | TRUE | TRUE | TRUE | TRUE | FALSE | 4 |
| atpI | TRUE | TRUE | TRUE | TRUE | TRUE | 5 |
| cbbx | TRUE | TRUE | TRUE | TRUE | FALSE | 4 |
| ccs1 | TRUE | TRUE | TRUE | TRUE | FALSE | 4 |
| ccsA | TRUE | TRUE | TRUE | TRUE | FALSE | 4 |
| chlB | TRUE | TRUE | TRUE | TRUE | FALSE | 4 |
| chlI | TRUE | TRUE | TRUE | TRUE | TRUE | 5 |
| chlL | TRUE | TRUE | TRUE | TRUE | TRUE | 5 |
| chlN | TRUE | TRUE | TRUE | TRUE | TRUE | 5 |
| clpC | TRUE | TRUE | TRUE | TRUE | TRUE | 5 |
| dnaB | TRUE | TRUE | TRUE | TRUE | FALSE | 4 |
| dnaK | TRUE | TRUE | TRUE | TRUE | FALSE | 4 |
| ftrB | TRUE | TRUE | TRUE | FALSE | FALSE | 3 |
| ftsH | TRUE | TRUE | TRUE | TRUE | TRUE | 5 |
| groEL | TRUE | TRUE | TRUE | TRUE | TRUE | 5 |
| ilvB | TRUE | TRUE | TRUE | TRUE | TRUE | 5 |
| ilvH | TRUE | TRUE | TRUE | TRUE | FALSE | 4 |
| petA | TRUE | TRUE | TRUE | TRUE | TRUE | 5 |
| petB | TRUE | TRUE | TRUE | TRUE | TRUE | 5 |
| petD | TRUE | TRUE | TRUE | TRUE | TRUE | 5 |
| petF | TRUE | TRUE | TRUE | TRUE | FALSE | 4 |
| petG | TRUE | TRUE | FALSE | FALSE | FALSE | 2 |
| petJ | TRUE | TRUE | TRUE | TRUE | TRUE | 5 |
| petM | FALSE | FALSE | FALSE | TRUE | FALSE | 1 |
| petN | FALSE | TRUE | FALSE | TRUE | FALSE | 2 |

| | | | | | | |
|---|---|---|---|---|---|---|
| psaA | TRUE | TRUE | TRUE | TRUE | TRUE | 5 |
| psaB | TRUE | TRUE | TRUE | TRUE | FALSE | 4 |
| psaC | TRUE | TRUE | TRUE | TRUE | FALSE | 4 |
| psaD | TRUE | TRUE | TRUE | TRUE | FALSE | 4 |
| psaE | TRUE | TRUE | TRUE | TRUE | FALSE | 4 |
| psaF | TRUE | TRUE | TRUE | TRUE | TRUE | 5 |
| psaI | TRUE | FALSE | FALSE | TRUE | FALSE | 2 |
| psaJ | TRUE | FALSE | TRUE | TRUE | FALSE | 3 |
| psaL | TRUE | TRUE | TRUE | TRUE | FALSE | 4 |
| psaM | TRUE | TRUE | FALSE | FALSE | FALSE | 2 |
| psb28 | TRUE | TRUE | TRUE | TRUE | FALSE | 4 |
| psbA | TRUE | TRUE | TRUE | TRUE | TRUE | 5 |
| psbB | TRUE | TRUE | TRUE | TRUE | TRUE | 5 |
| psbC | TRUE | TRUE | TRUE | TRUE | TRUE | 5 |
| psbD | TRUE | TRUE | TRUE | TRUE | TRUE | 5 |
| psbE | TRUE | TRUE | TRUE | TRUE | FALSE | 4 |
| psbF | TRUE | TRUE | TRUE | TRUE | FALSE | 4 |
| psbH | FALSE | TRUE | TRUE | FALSE | FALSE | 2 |
| psbI | TRUE | TRUE | TRUE | TRUE | TRUE | 5 |
| psbJ | TRUE | TRUE | TRUE | FALSE | FALSE | 3 |
| psbK | TRUE | TRUE | TRUE | TRUE | TRUE | 5 |
| psbL | TRUE | TRUE | TRUE | FALSE | FALSE | 3 |
| psbN | TRUE | FALSE | TRUE | FALSE | TRUE | 3 |
| psbT | TRUE | FALSE | FALSE | TRUE | FALSE | 2 |
| psbV | TRUE | TRUE | TRUE | TRUE | TRUE | 5 |
| psbX | TRUE | TRUE | TRUE | FALSE | FALSE | 3 |
| psbY | TRUE | TRUE | TRUE | TRUE | FALSE | 4 |
| rbcL | TRUE | TRUE | TRUE | TRUE | FALSE | 4 |
| rbcR | TRUE | TRUE | TRUE | FALSE | FALSE | 3 |
| rbcS | TRUE | TRUE | TRUE | TRUE | TRUE | 5 |
| rpl11 | TRUE | TRUE | TRUE | TRUE | FALSE | 4 |
| rpl12 | TRUE | TRUE | TRUE | TRUE | TRUE | 5 |
| rpl13 | TRUE | TRUE | TRUE | TRUE | FALSE | 4 |
| rpl14 | TRUE | TRUE | TRUE | TRUE | FALSE | 4 |
| rpl16 | TRUE | TRUE | TRUE | TRUE | TRUE | 5 |
| rpl18 | TRUE | TRUE | TRUE | TRUE | FALSE | 4 |
| rpl19 | TRUE | TRUE | FALSE | TRUE | FALSE | 3 |
| rpl1 | TRUE | TRUE | TRUE | TRUE | TRUE | 5 |

| | | | | | | |
|---|---|---|---|---|---|---|
| rpl20 | TRUE | TRUE | TRUE | TRUE | FALSE | 4 |
| rpl21 | TRUE | TRUE | FALSE | TRUE | FALSE | 3 |
| rpl22 | TRUE | TRUE | TRUE | TRUE | TRUE | 5 |
| rpl23 | TRUE | TRUE | FALSE | TRUE | FALSE | 3 |
| rpl24 | TRUE | TRUE | TRUE | FALSE | FALSE | 3 |
| rpl27 | FALSE | FALSE | FALSE | TRUE | FALSE | 1 |
| rpl29 | TRUE | TRUE | TRUE | FALSE | FALSE | 3 |
| rpl2 | TRUE | TRUE | TRUE | TRUE | FALSE | 4 |
| rpl31 | TRUE | TRUE | TRUE | TRUE | FALSE | 4 |
| rpl32 | TRUE | TRUE | TRUE | FALSE | FALSE | 3 |
| rpl33 | TRUE | TRUE | TRUE | TRUE | TRUE | 5 |
| rpl34 | TRUE | TRUE | TRUE | TRUE | FALSE | 4 |
| rpl35 | TRUE | TRUE | TRUE | TRUE | TRUE | 5 |
| rpl36 | TRUE | TRUE | TRUE | FALSE | FALSE | 3 |
| rpl3 | TRUE | TRUE | TRUE | TRUE | TRUE | 5 |
| rpl4 | TRUE | TRUE | TRUE | TRUE | FALSE | 4 |
| rpl5 | TRUE | TRUE | FALSE | TRUE | TRUE | 4 |
| rpl6 | TRUE | TRUE | TRUE | TRUE | TRUE | 5 |
| rpl9 | TRUE | TRUE | TRUE | TRUE | TRUE | 5 |
| rpoA | TRUE | TRUE | TRUE | TRUE | TRUE | 5 |
| rpoB | TRUE | TRUE | TRUE | TRUE | TRUE | 5 |
| rpoC1 | TRUE | TRUE | TRUE | TRUE | FALSE | 4 |
| rpoC2 | TRUE | TRUE | TRUE | TRUE | FALSE | 4 |
| rps10 | TRUE | TRUE | TRUE | TRUE | FALSE | 4 |
| rps11 | TRUE | TRUE | TRUE | TRUE | FALSE | 4 |
| rps12 | TRUE | TRUE | TRUE | TRUE | FALSE | 4 |
| rps13 | TRUE | TRUE | TRUE | TRUE | TRUE | 5 |
| rps14 | TRUE | TRUE | TRUE | TRUE | FALSE | 4 |
| rps16 | TRUE | TRUE | TRUE | FALSE | FALSE | 3 |
| rps17 | TRUE | TRUE | TRUE | TRUE | TRUE | 5 |
| rps18 | TRUE | TRUE | FALSE | TRUE | FALSE | 3 |
| rps19 | TRUE | TRUE | TRUE | TRUE | FALSE | 4 |
| rps1 | TRUE | TRUE | TRUE | TRUE | TRUE | 5 |
| rps20 | TRUE | TRUE | TRUE | TRUE | TRUE | 5 |
| rps2 | TRUE | TRUE | TRUE | TRUE | TRUE | 5 |
| rps3 | TRUE | TRUE | TRUE | TRUE | FALSE | 4 |
| rps4 | TRUE | TRUE | TRUE | TRUE | FALSE | 4 |
| rps5 | TRUE | TRUE | TRUE | TRUE | TRUE | 5 |

| | | | | | | |
|---|---|---|---|---|---|---|
| rps7 | TRUE | TRUE | TRUE | FALSE | FALSE | 3 |
| rps8 | TRUE | TRUE | TRUE | TRUE | FALSE | 4 |
| rps9 | TRUE | TRUE | TRUE | FALSE | FALSE | 3 |
| secA | TRUE | TRUE | TRUE | TRUE | FALSE | 4 |
| secY | TRUE | TRUE | TRUE | TRUE | TRUE | 5 |
| sufB | TRUE | TRUE | TRUE | TRUE | TRUE | 5 |
| sufC | TRUE | TRUE | TRUE | TRUE | TRUE | 5 |
| tatC | TRUE | TRUE | TRUE | TRUE | TRUE | 5 |
| thiG | TRUE | TRUE | TRUE | TRUE | FALSE | 4 |
| this | TRUE | TRUE | TRUE | FALSE | FALSE | 3 |
| tsf | TRUE | TRUE | TRUE | TRUE | FALSE | 4 |
| tufA | TRUE | TRUE | TRUE | TRUE | TRUE | 5 |
| ycf12 | TRUE | FALSE | FALSE | FALSE | FALSE | 1 |
| ycf19 | FALSE | TRUE | TRUE | TRUE | TRUE | 4 |
| ycf33 | TRUE | TRUE | TRUE | TRUE | FALSE | 4 |
| ycf34 | TRUE | TRUE | FALSE | TRUE | FALSE | 3 |
| ycf35 | TRUE | TRUE | TRUE | TRUE | FALSE | 4 |
| ycf37 | TRUE | TRUE | TRUE | TRUE | FALSE | 4 |
| ycf39 | TRUE | TRUE | TRUE | TRUE | FALSE | 4 |
| ycf3 | TRUE | TRUE | TRUE | FALSE | FALSE | 3 |
| ycf41 | TRUE | TRUE | TRUE | TRUE | TRUE | 5 |
| ycf42 | TRUE | FALSE | TRUE | TRUE | FALSE | 3 |
| ycf46 | TRUE | TRUE | TRUE | TRUE | FALSE | 4 |
| ycf47 | TRUE | TRUE | FALSE | FALSE | FALSE | 2 |
| ycf4 | TRUE | TRUE | TRUE | TRUE | FALSE | 4 |
| ycf65 | TRUE | TRUE | TRUE | FALSE | FALSE | 3 |
| ycf66 | TRUE | TRUE | TRUE | FALSE | FALSE | 3 |
| Total supporting genes | 130 | 127 | 120 | 112 | 51 | |
