# Supplementary material for: Plastid and mitochondrial genomes of Coccophora langsdorfii (Fucales, Phaeophyceae) and the utility of molecular markers
Source: PLoS One. 2017 Nov 2;12(11):e0187104. doi: 10.1371/journal.pone.0187104 (PMC5695614; doi:10.1371/journal.pone.0187104)
Supplement: S3 Table — TRUE indicates that the node is supported with above 50% Maximum Likelihood Bootstrap value. Underlined genes are more than 300 amino acids. (PDF) [file pone.0187104.s009.pdf]

S3 Table. Summary of the accordance between the mitochondria encoded single gene trees topologies and the phylogeny of the Phaeophyceae. TRUE indicates that the node is supported with above 50% Maximum Likelihood Bootstrap value. Underlined genes are more than 300 amino acids.

gene	A	B	C	D	E	F	G	H	I	J	K	L	M	N	O	P	Total supported nodes
atp6	TRUE	TRUE	TRUE	FALSE	TRUE	TRUE	TRUE	TRUE	FALSE	TRUE	TRUE	FALSE	TRUE	FALSE	TRUE	FALSE	11
atp8	TRUE	TRUE	TRUE	FALSE	TRUE	FALSE	FALSE	TRUE	FALSE	TRUE	TRUE	TRUE	TRUE	FALSE	TRUE	TRUE	11
atp9	TRUE	TRUE	TRUE	FALSE	FALSE	FALSE	FALSE	FALSE	FALSE	FALSE	TRUE	FALSE	FALSE	FALSE	TRUE	TRUE	6
cob	TRUE	TRUE	TRUE	TRUE	TRUE	TRUE	TRUE	TRUE	TRUE	TRUE	TRUE	TRUE	TRUE	TRUE	TRUE	TRUE	16
cox1	TRUE	TRUE	TRUE	TRUE	TRUE	TRUE	TRUE	TRUE	FALSE	TRUE	TRUE	TRUE	FALSE	FALSE	TRUE	TRUE	13
cox2	TRUE	TRUE	TRUE	TRUE	TRUE	FALSE	FALSE	FALSE	FALSE	TRUE	TRUE	TRUE	FALSE	FALSE	TRUE	TRUE	10
cox3	TRUE	TRUE	TRUE	TRUE	TRUE	TRUE	TRUE	TRUE	FALSE	TRUE	TRUE	TRUE	TRUE	FALSE	FALSE	TRUE	13
nad1	TRUE	TRUE	TRUE	TRUE	TRUE	TRUE	TRUE	TRUE	FALSE	TRUE	TRUE	TRUE	FALSE	FALSE	TRUE	TRUE	13
nad11	TRUE	FALSE	TRUE	TRUE	TRUE	TRUE	TRUE	TRUE	FALSE	TRUE	TRUE	TRUE	FALSE	FALSE	FALSE	FALSE	10
nad2	TRUE	TRUE	TRUE	TRUE	TRUE	FALSE	TRUE	TRUE	FALSE	TRUE	TRUE	TRUE	TRUE	FALSE	TRUE	TRUE	13
nad3	TRUE	TRUE	TRUE	TRUE	TRUE	FALSE	FALSE	FALSE	FALSE	TRUE	TRUE	TRUE	FALSE	FALSE	TRUE	TRUE	10
nad4	TRUE	TRUE	TRUE	TRUE	TRUE	FALSE	FALSE	TRUE	TRUE	TRUE	TRUE	TRUE	TRUE	FALSE	FALSE	FALSE	11
nad4L	TRUE	FALSE	TRUE	TRUE	FALSE	FALSE	FALSE	TRUE	FALSE	TRUE	FALSE	TRUE	FALSE	FALSE	TRUE	TRUE	8
nad5	TRUE	TRUE	TRUE	TRUE	TRUE	TRUE	TRUE	TRUE	FALSE	TRUE	TRUE	TRUE	TRUE	FALSE	TRUE	TRUE	14
nad6	TRUE	TRUE	TRUE	TRUE	TRUE	FALSE	FALSE	FALSE	FALSE	TRUE	TRUE	TRUE	FALSE	FALSE	TRUE	TRUE	10
nad7	TRUE	TRUE	TRUE	TRUE	TRUE	FALSE	FALSE	TRUE	FALSE	TRUE	TRUE	TRUE	TRUE	FALSE	TRUE	FALSE	11
nad9	TRUE	TRUE	TRUE	FALSE	TRUE	FALSE	FALSE	FALSE	FALSE	TRUE	FALSE	TRUE	TRUE	FALSE	TRUE	TRUE	9
rpl14	TRUE	TRUE	TRUE	FALSE	TRUE	TRUE	TRUE	TRUE	FALSE	TRUE	TRUE	TRUE	FALSE	FALSE	TRUE	TRUE	12
rpl16	TRUE	FALSE	FALSE	FALSE	FALSE	TRUE	FALSE	TRUE	TRUE	TRUE	FALSE	TRUE	FALSE	FALSE	TRUE	TRUE	8
rpl2	TRUE	FALSE	TRUE	TRUE	TRUE	FALSE	FALSE	TRUE	FALSE	TRUE	TRUE	TRUE	TRUE	FALSE	TRUE	TRUE	11

rpl31	TRUE	TRUE	TRUE	TRUE	TRUE	FALSE	FALSE	TRUE	FALSE	TRUE	TRUE	TRUE	TRUE	TRUE	TRUE	TRUE	13
rpl5	TRUE	TRUE	TRUE	FALSE	TRUE	FALSE	FALSE	FALSE	FALSE	FALSE	FALSE	TRUE	FALSE	FALSE	TRUE	TRUE	7
rpl6	TRUE	TRUE	TRUE	TRUE	TRUE	FALSE	TRUE	TRUE	FALSE	TRUE	TRUE	TRUE	TRUE	FALSE	TRUE	TRUE	13
rps10	TRUE	TRUE	TRUE	TRUE	TRUE	FALSE	FALSE	FALSE	FALSE	FALSE	TRUE	TRUE	TRUE	FALSE	TRUE	TRUE	10
rps11	TRUE	TRUE	TRUE	TRUE	TRUE	FALSE	FALSE	TRUE	FALSE	TRUE	FALSE	TRUE	FALSE	FALSE	TRUE	TRUE	10
rps12	TRUE	TRUE	TRUE	TRUE	TRUE	FALSE	FALSE	FALSE	FALSE	TRUE	TRUE	TRUE	TRUE	FALSE	TRUE	TRUE	11
rps13	TRUE	TRUE	TRUE	FALSE	TRUE	FALSE	FALSE	FALSE	FALSE	FALSE	FALSE	FALSE	FALSE	FALSE	TRUE	TRUE	6
rps14	TRUE	TRUE	TRUE	TRUE	TRUE	FALSE	FALSE	FALSE	FALSE	FALSE	TRUE	TRUE	TRUE	TRUE	TRUE	TRUE	11
rps19	TRUE	FALSE	TRUE	TRUE	TRUE	FALSE	FALSE	FALSE	FALSE	TRUE	FALSE	TRUE	TRUE	FALSE	FALSE	FALSE	7
rps2	TRUE	TRUE	TRUE	TRUE	TRUE	FALSE	FALSE	FALSE	FALSE	FALSE	TRUE	TRUE	FALSE	FALSE	TRUE	TRUE	9
rps3	TRUE	TRUE	TRUE	TRUE	TRUE	FALSE	TRUE	TRUE	FALSE	TRUE	TRUE	TRUE	FALSE	FALSE	TRUE	TRUE	12
rps4	TRUE	TRUE	TRUE	TRUE	TRUE	TRUE	TRUE	TRUE	FALSE	TRUE	FALSE	TRUE	TRUE	FALSE	TRUE	TRUE	13
rps7	TRUE	TRUE	TRUE	TRUE	TRUE	TRUE	TRUE	TRUE	FALSE	TRUE	TRUE	TRUE	TRUE	FALSE	TRUE	TRUE	14
rps8	TRUE	TRUE	TRUE	TRUE	TRUE	FALSE	FALSE	TRUE	FALSE	TRUE	TRUE	TRUE	FALSE	FALSE	TRUE	TRUE	11
tatC	TRUE	TRUE	TRUE	TRUE	TRUE	FALSE	FALSE	FALSE	TRUE	FALSE	TRUE	TRUE	TRUE	FALSE	TRUE	TRUE	11
Total supporting genes	35	30	34	27	32	11	13	22	4	28	27	32	19	3	31	30	
