# Supplementary material for: Plastid and mitochondrial genomes of Coccophora langsdorfii (Fucales, Phaeophyceae) and the utility of molecular markers
Source: PLoS One. 2017 Nov 2;12(11):e0187104. doi: 10.1371/journal.pone.0187104 (PMC5695614; doi:10.1371/journal.pone.0187104)
Supplement: S4 Fig — White characters in a red box represent strict identity between all sequences. Red characters represent similarity in a group of sequences. Blue frame represent a conserved region. Green highlight represent the in frame insertion found in the Fucales, Desmarestiales, Ectocarpales and Laminariales. (PDF) [file pone.0187104.s004.pdf]

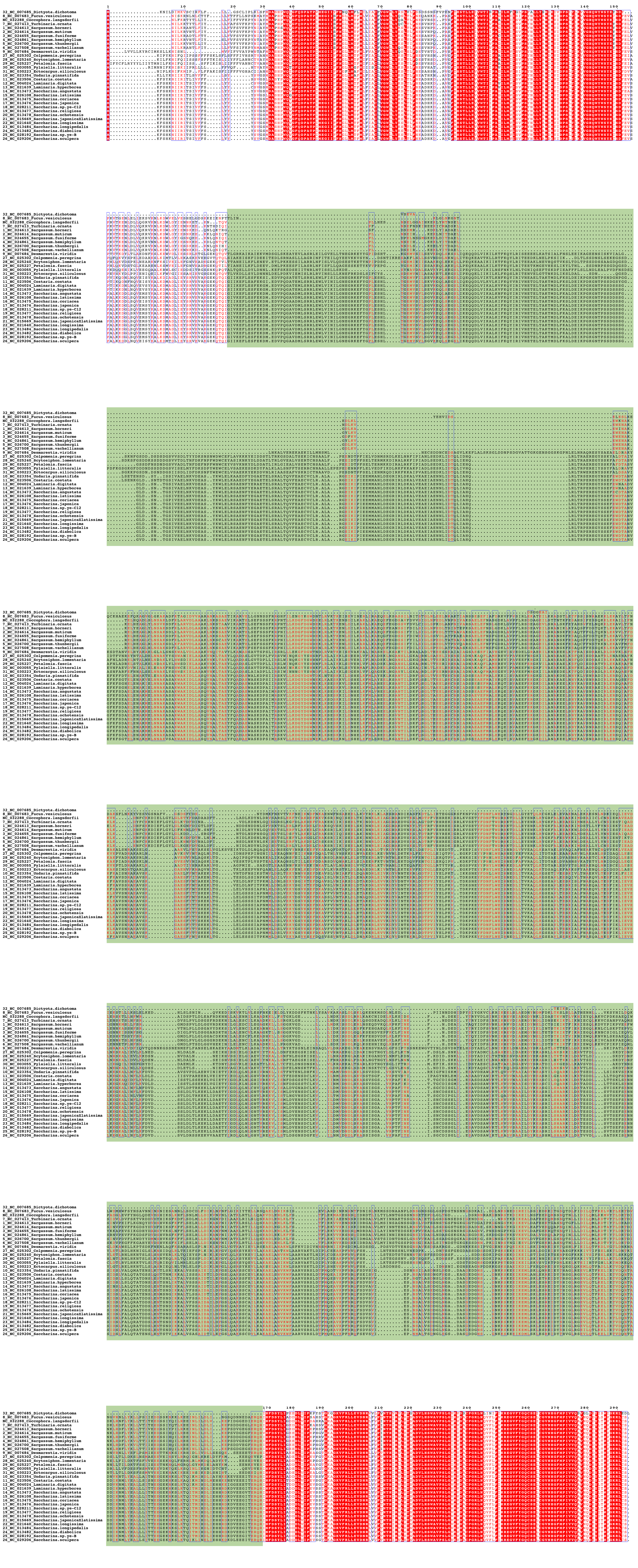

S4 Fig. Aligned sequences of the cox2 gene in the class Phaeophyceae. White characters in a red box represent strict identity between all sequences. Red characters represent similarity in a group of sequences. Blue frame represent a conserved region. Green highlight represent the in frame insertion found in the Fucales, Desmarestiales, Ectocarpales and Laminariales.
